# New insights about host response to smallpox using microarray data

**DOI:** 10.1186/1752-0509-1-38

**Published:** 2007-08-24

**Authors:** Gustavo H Esteves, Ana CQ Simoes, Estevao Souza, Rodrigo A Dias, Raydonal Ospina, Thiago M Venancio

**Affiliations:** 1 BIOINFO, Instituto de Matemática e Estatística, Universidade de São Paulo, Brazil; 2 Laboratório de Bioinformática, Departamento de Bioquímica, Instituto de Química, Universidade de São Paulo, Brazil; 3 Departamento de Estatística, Instituto de Matemática e Estatística, Universidade de São Paulo, Brazil; 4 Departamento de Matemática e Estatística, Centro de Ciências e Tecnologia, Universidade Estadual da Paraíba, Brazil

## Abstract

**Background:**

Smallpox is a lethal disease that was endemic in many parts of the world until eradicated by massive immunization. Due to its lethality, there are serious concerns about its use as a bioweapon. Here we analyze publicly available microarray data to further understand survival of smallpox infected macaques, using systems biology approaches. Our goal is to improve the knowledge about the progression of this disease.

**Results:**

We used KEGG pathways annotations to define groups of genes (or modules), and subsequently compared them to macaque survival times. This technique provided additional insights about the host response to this disease, such as increased expression of the cytokines and ECM receptors in the individuals with higher survival times. These results could indicate that these gene groups could influence an effective response from the host to smallpox.

**Conclusion:**

Macaques with higher survival times clearly express some specific pathways previously unidentified using regular gene-by-gene approaches. Our work also shows how third party analysis of public datasets can be important to support new hypotheses to relevant biological problems.

## Background

Large scale gene expression analysis with microarray technology is expanding and generating a large amount of high quality, publicly available data. In the present work we analyzed a dataset derived from monkeys infected by smallpox, published by Rubins et al [1]. Smallpox is a lethal disease that was endemic in many parts of the world until eradicated by a massive immunization program developed by the World Health Organization. Its fatality rate was estimated to be 30%, and the survivors often had disfiguring scars [2].

There are serious concerns about the use of smallpox as a bioweapon [3,4]. Recently, some health care workers were vaccinated by the UK government for the analysis of antibody responses [5]. Pox viruses display unique abilities to interfere with the host immune system, producing immune modulators [6] and there are at least 16 viral genes involved in combating the host immune response [7]. The original study's goal was to analyze the evolution of the gene expression of the peripheral blood cells of variola-infected monkeys, so as to clarify the biological processes associated with host-pathogen interactions [1].

Among the important results was the absence of a TNF-*α*/NF-*κ*B-activated transcriptional mechanism during systemic infection, which could suggest that variola interaction with the mammalian host immune system may be ablative to this response [6].

Given the importance of this dataset, we have analyzed the data with systems biology approaches considering the expression profiles of gene groups instead of individual genes. These approaches are more powerful, less vulnerable to common experimental variation and usually more advantageous for obtaining biologically meaningful results.

## Results and discussion

### Active Modules

The goal of our analysis was to identify differentially expressed modules in monkeys with early death, i.e., died in the first three days, and monkeys with a later death (ED and LD, respectively). Our hypothesis is that these modules play crucial roles in the host response to smallpox infection. The gene groups were defined according to the KEGG database (updated on 04/17/2006) [8], as described in the Methods section.

Initially, this analysis was based on the ED and LD monkey groups using all KEGG Orthology (KO) categories (at level 4), indicating three highly active modules in LD monkeys: Cytokines and Cell Adhesion Molecules (CAMs), which are consistent with the classical model of immune and inflammatory responses, where an activation and proliferation of leukocytes take place. The Alzheimer's Disease module also appeared as differentially activated, most likely due to its hybrid composition (20/22 genes that compose the AD module are present in the microarray platform used by Rubins et al [1], and 14/20 are also members of other modules).

**Figure 1:**
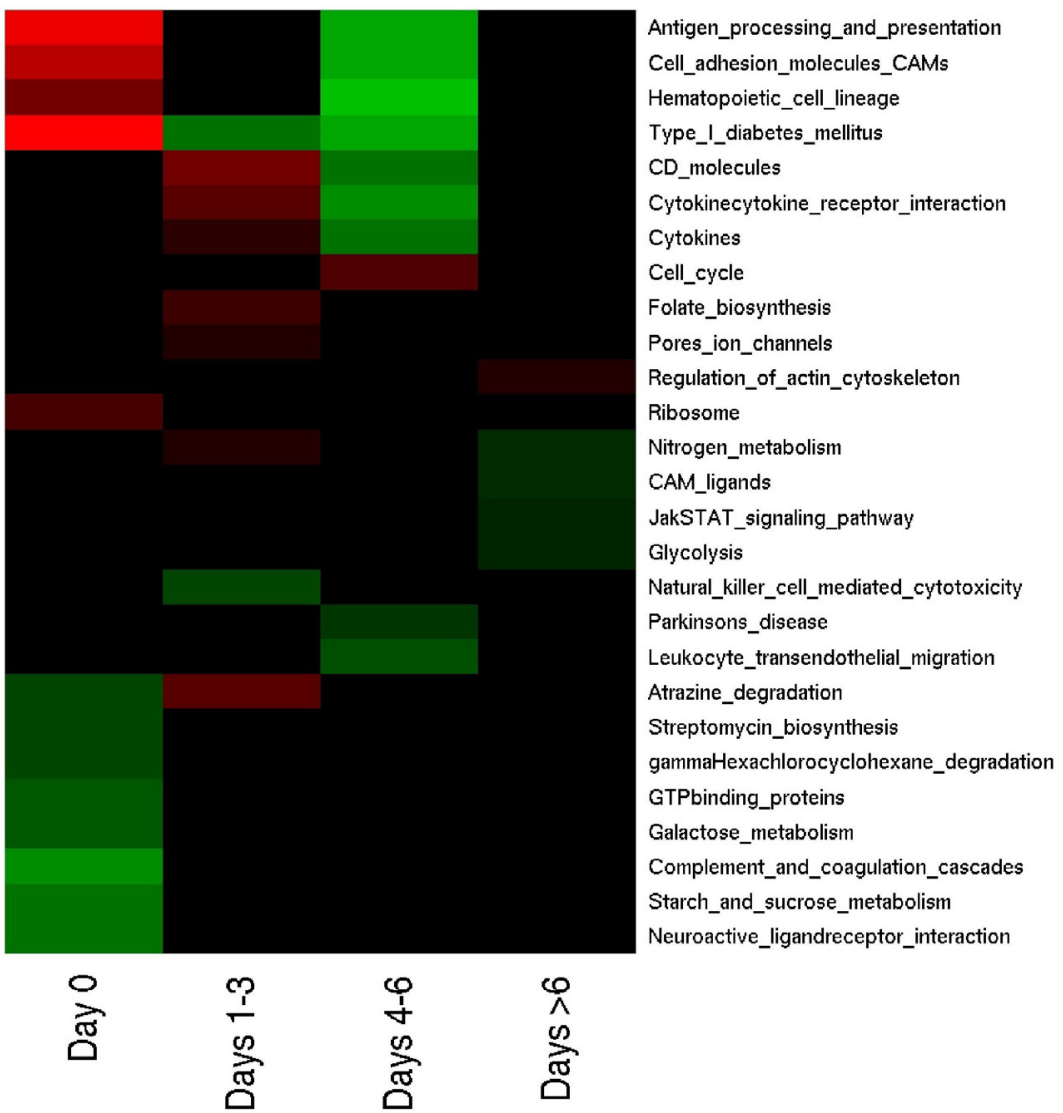
**Active module analysis**. Heat map representation of gene groups (modules) with significant alteration (*p *< 0.05) are shown. The red and green colors represent positive (activation) and negative (repression) regulation, respectively.

In addition to the ED and LD analysis, modules were also analyzed during the disease course. We have found 27 modules presenting differential activation in at least one point (Figure 1). Several of those modules are related to immune response, like CD molecules, CAM ligands, antigen processing and presentation and the previously considered activated in a late death Cytokines and Cell adhesion molecules.

A novel finding highlighted by our systems biology approach for all macaques (Figure 1) was an altered expression in the Natural Killer (NK) mediated cytotoxicity module. NK cells play a major role in the host-rejection of virally infected cells. Due to their strong cytolytic activity and the potential for auto-reactivity, they are tightly regulated. The activation of NK cells requires the action of proinflammatory cytokines in combination with cell surface receptors [9]. Under normal conditions, inhibitory NK cell receptors engage MHC class I (MHC-I) molecules. Such inhibitory activity normally predominates over that provided by the activating receptors, which bind to ligands expressed on virus-infected and other stressed cells [10]. Cells with normal levels of MHC-I are generally protected from NK cell-mediated lysis. Virally infected cells often express reduced levels of MHC-I and this inability to engage the inhibitory receptors makes the cells susceptible to NK cell attack [10]. The down regulation of Natural killer cell mediated cytotoxicity module reported here could indicate a susceptibility to a NK cell mediated lysis (days 1–3, Figure 1).

The contributions of individual molecules for each significantly altered module are available in the additional material [see Additional files 1 and 2], represented by scores and the respective *p*-values.

### Relevance Networks (RNs)

To understand the profiles of interaction between the genes of the groups studied, we used a procedure known as relevance networks (RNs), initially proposed by Butte et al [11]. Interaction here represents significant correlation between two variables (i.e. molecules), which may (but not necessarily will) physically interact with each other.

**Figure 2:**
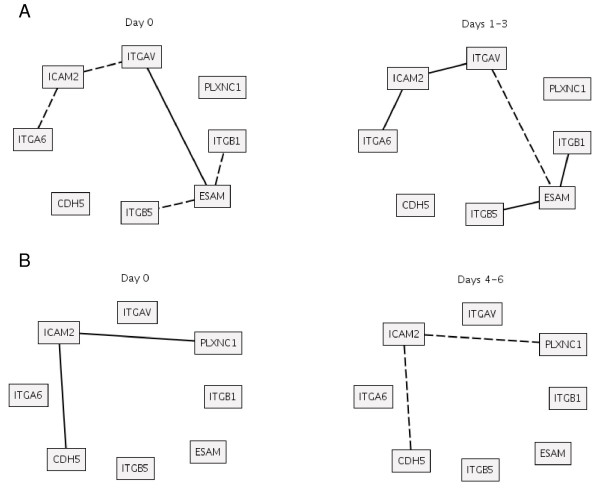
**Relevance networks analysis**. Relevance networks (*p *< 10^-3^). Changes in correlation between genes at day 0 (left panel) and days 1–3 (right panel) (**A**) and day 0 (left panel) versus days 4–6 (right panel) (**B**). Solid and dashed lines represent positive and negative correlation values, respectively.

Cytokines and cell adhesion molecules (CAMs) modules were highly expressed in LD monkeys in the early phase of the infection (Figure 1) and were further explored in more detail by using the RN approach. A metamodule (CAMs and ECM receptors combined) was created to gain a better understanding of leukocyte migration to the infection locus. The connections between ICAM2 and ITGAV and ITGA6 shows a significant change (p value < 10^-3^) when comparing day 0 versus the earliest infection groups (days 1–3), see Figure 2A.

To establish a successful infection, pox viruses affect the release of immune modulators by the infected cells, so it is crucial to understand the dynamics of the host immune system [6]. The population of the immune cells varies during the infection, as do their exposed membrane proteins. Therefore, the Relevance Networks approach can reveal a more detailed perspective on the dynamics of the molecules involved in the disease. A better assessment of the variation of those molecules during the onset of the infection could give a better understanding of the host response to smallpox, and ultimately of what may be the key factors that set the 30% mortality rate.

To reach the infection focus and kill the pathogen, the immune cells have to adhere and overpass the endothelium. These events are possible due to the interaction between the integrins and the extra cellular matrix receptors and are modulated by cytokines. More specifically, ESAM is an endothelial cell adhesion molecule and ITGAV, ITGB1 and ITGB5 are integrins, which are heterodimeric integral membrane proteins composed of an alpha chain and a beta chain. ITGAV encodes an alpha chain; ITGB1 and ITGB5 encode beta chain integrins, known to participate in the cell-surface mediated signaling. ICAM2 is an intercellular adhesion molecule that promotes an inflammatory response and the activation of AKT (a kinase). AKT phosphorylates components that block the apoptosis pathway [12,13]. To explore further the graphs produced by RNs for the ECM receptors + CAMs the relations concerning ICAM2 and ESAM are detailed. The complete network is available in the additional material [see Additional files 3 and 4]. RNs of day 0 and days 1–3 show statistically significant alterations in the interactions between ICAM2 and the integrins ITGAV and ITGA6 (Figure 2A), which are important extracellular matrix receptors, involved in the adhesion, migration, differentiation, survival and proliferation of cells and cytoskeleton organization. Day 0 versus days 4–6 RNs revealed a significant relation between ICAM2 and other molecules that play crucial role in immune response against pathogens, PLXNC1 [14] and CDH5 [15] (Figure 2B). The relevance networks produced for cytokine group is available in the additional material [see Additional file 5].

ESAM shows a strong relation with ITGAV, ITGB1 and ITGB5 with variable patterns along the time course of infection. The quartet of ESAM, ITGAV, ITGB1 and ITGB5 reveals the relationship between alpha and beta chains in the composition of integrins and the relation of such heterodimeric proteins with ESAM. It is worth mentioning that ITGB3, ITGB6 and ITGB8, elements that interact with ITGAV, are present in the microarray platform design, but were excluded from analysis in the filtering steps (see Methods section).

Active modules and the RNs results pointed out drastic changes in the expression levels and interactions of important genes involved in essential cellular processes of the host response, such as cellular adhesion, apoptosis and immune response. These results constitute interesting new biological hypotheses about smallpox virus infection that could be further explored by expert virology groups.

### Overlapping with Rubins et al and Jahrling et al

The data analyzed in our manuscript was previously analyzed by Rubins et al [1] and Jahrling et al [16]. The former focused on the microarray data and the later on the clinical findings. Rubins et al [1] reported the expression profile in IFN response, cell cycle/proliferation response, TNF-*α*/NF-*κ*B regulated genes, dose response and lymphocyte and Ig responses. Meanwhile Jahrling et al [16] discuss the hemorrhagic diathesis, cytokines activation (IL6, IL8, INFG, CCL2 and CCL4) and lymphocyte depletion.

Rubins et al [1] performed a detailed analysis of IFN response genes. The KEGG module of cytokines includes IL6, IL8, IFN -*α*, -*β *and -*γ*, CCL2 (MCP-1), CCL4 (MIP1*β*), TNF-*α *and NF – *κ*B. The influence of each molecule in the modules activation was also computed [see Additional files 1 and 2]. Once KEGG separates the molecules into functional classes, it is not possible to define cascading effects, like Rubins et al did [1]. Despite this fact, we manually compiled a module with the IFN-responsive genes detailed by Rubins et al [1] (in Figure 2A). For the ED and LD analysis, this module is significantly down regulated in monkeys that died early and up regulated in those which died later (see Methods for details). In addition, for the time course analysis, the IFN-responsive module is down regulated for day 0 and up regulated for days 1–3 and inactive for the other days (data not shown). Therefore, our results suggest that activation of IFN-responsive genes may play an important role in a successful immune response upon infection, as previously reported by Rubins et al [1].

The decrease in the relative abundance of B cells and MHC II reported by Rubins et al [1] are corroborated by the profile of module Antigen processing and presentation (Figure 1). The Cell cycle, Complement and coagulation cascades, Cytokines and Hematopoietic cell lineage modules identified here were also reported by Rubins et al [1] as differentially expressed using gene by gene approaches.

Altered D-dimer levels were interestingly investigated by Jahrling et al [16], which reflect the serious influence of smallpox virus inhibitors in the complement and coagulation cascades. Our active modules are also in agreement with such findings. Our results suggest an unbalanced cytokine levels, which is strongly associated with sepsis and organ failure death in infected animals.

In addition, we have also observed alterations in the hematopoietic cell lineage module which could be a consequence of the influx of immature leukocytes into the bloodstream, particularly neutrophils (aka "left shift", using medical jargon).

Rubins et al [1] observed a considerable depletion of T cells, and a large number of apoptotic cells in the splenic periarterial lymphatic sheath, possibly due to the viral replication in antigen presenting cells in the lymphatic tissues, which could lead to apoptosis in the lymphocytes. The lysis of vulnerable cells induced by NK cells (described earlier) and the down regulation of the NK cell mediated cytotoxicity module supports such hypothesis.

## Conclusion

The usage of active modules pointed out the major role of the cytokines and ECM receptors in an effective host response. We have also pointed out that the population of the ECM receptors and cytokines significantly changes during the infection, possible due to a host response to eliminate the virus. In order to have a closer perspective of the dynamics of the molecules involved in this response we used the Relevance Networks. Despite the need of an experimental validation of these new biological hypotheses, one of our goals was to exemplify and stress the importance of public microarray datasets analysis by using different and novel bioinformatics models and thus expand knowledge and information about important biological problems.

## Methods

### Data description

In this work we used cDNA microarray data containing 37,632 elements that represents approximately 11,000 unique genes and approximately 12,000 genes without identification [1,17]. The slides were produced with human cDNA clones and the test samples were extracted from primate models (cynomolgus macaques). The samples of interest were always labeled with Cy5 and the reference samples were labeled with Cy3. The original dataset was obtained from the SMD [18,19] and is associated with the Experimenter and ExptDate keys "*KRUBINS" *and "2001-08-05", respectively. In this work, only data associated to studies 1 (Harper or India 7124 variola strains through a combination of aerosol exposure [5 × 108 plaque-forming units (pfu)] and high-titer i.v. inoculation [109 pfu]) and 2 (India 7124 strain via i.v. route alone [109 pfu]) [1] were used. Our goal is to analyze the host response to a successful infection; therefore the third study (varied viral dose) data was not used here.

**Figure 3:**
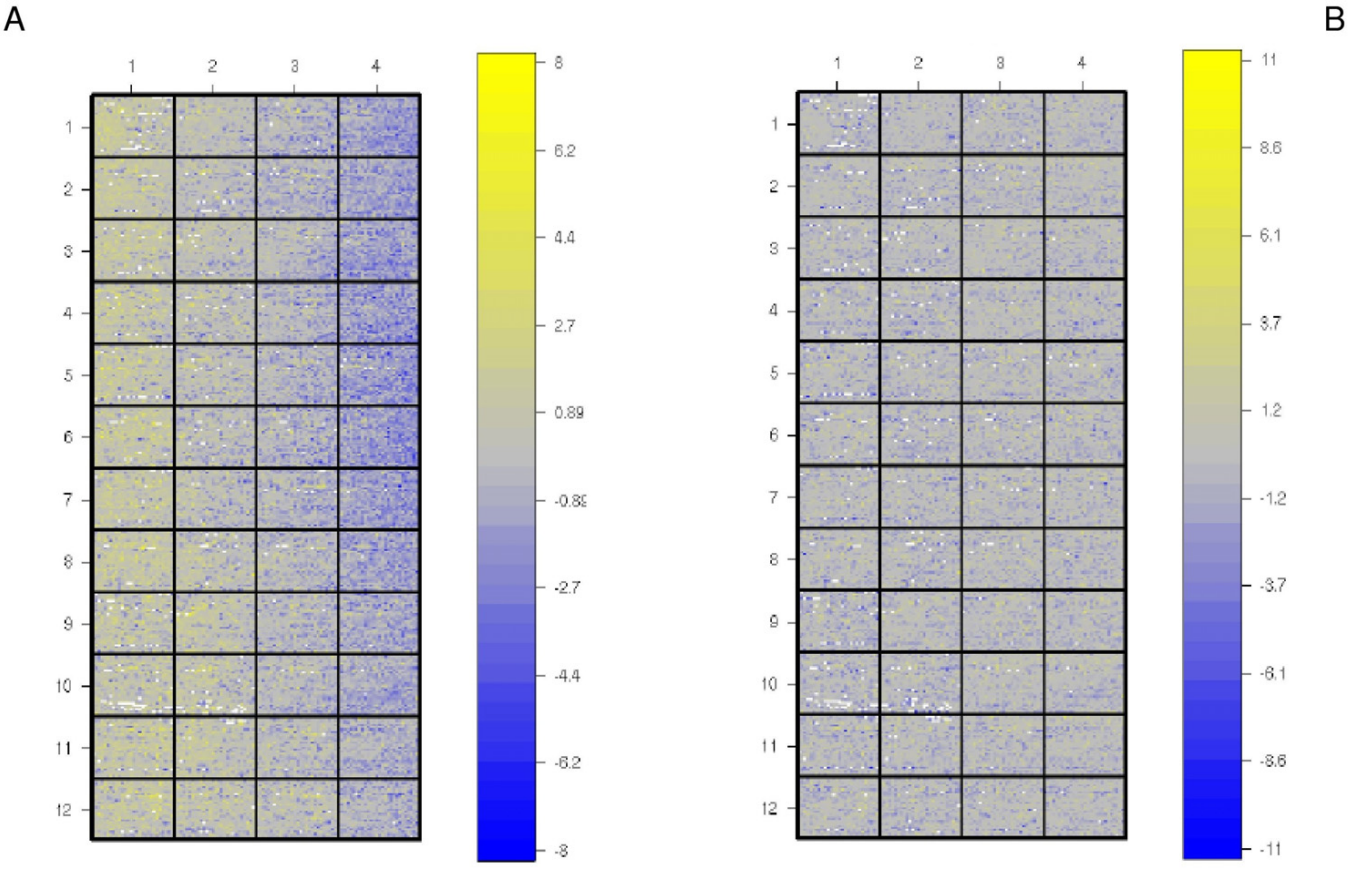
**OLIN normalization**. OLIN normalization method proposed by Futschik and Crompton [20]. **A- **The spatial bias effect found. This effect was noted in most of the chips; **B- **The same slide after OLIN normalization.

The raw data was submitted to exploratory analyses. The main problem found was a bias of log ratio values from left to right in the majority of the chips, see Figure 3A. To address this problem, we used a normalization procedure proposed by Futschik and Crompton [20] known as OLIN. The OLIN method has some clear advantages: (i) it relies on strong statistical formalism; (ii) has the capacity to remove spatial and intensity (systematic) errors in microarray data, which is the main problem we found in the original dataset; (iii) Futschick and Crompton [20] precisely addressed the importance of optimization of model parameters on microarray data normalization (instead of simply accepting the method default values); (iv) the method performance was conveniently compared to other proposed methods [20]. Basically, the effect of log ratio values in relation to mean expression values (M and A, respectively) was is estimated using LOWESS regression [21]with an optimization step for the selection of the parameter *α*_*A *_that control the smoothing of the regression. Subsequently, a new LOWESS regression was performed with log ratio values based on the x and y directions on the slide, and again, the optimization step of the parameters *α*_*x *_and *α*_*y *_was performed. This model successfully corrected the spatial-dependent artifact observed in the dataset (Figure 3B). Also, in a filtering step, we used only spots with signal intensity greater than background intensity in any sample and all spots with signal intensity greater than 2.5 times background intensity in 80% of the samples.

### Active modules

In order to find significantly altered gene groups we used the "active modules" approach, originally proposed by Segal et al [22]. The rationale of this method is to evaluate differential activation or repression of gene modules, which represent the biological processes.

The first step was to organize the data in a matrix *E*_*ij*_, *i *= 1,...,*n*_*s*_, *j *= 1,...,*n*_*l*_, where *e*_*ij *_represents the gene *i *in the sample *j*. In the present study, 199 groups were selected according to the level 4 of KEGG [8] (updated on 04/17/2006) [see Additional file 6], so each group has *n*_*g *_genes, *g *= 1,...,199.

After the data normalization described earlier, expression values were standardized by subtracting the average expression calculated over all the arrays. This process adjusted the mean values of each gene to zero, which were then used to build a discrete version of *E*_*ij*_, named *E*_ij_*, by applying the indicator function *e*_ij_* = I(*e*_*ij *_≥ 1) - I(*e*_*ij*_≤-1) for all *i *and *j *(an indicator function assumes value 0 or 1 for false or true, respectively). Thus, *E*_ij_* is a matrix where each element can assume the discrete values 1 (induction), 0 (equal expression) or -1(repression).

Using the matrix *E*_ij_* the following operations are performed:

q1j=∑i=1nsI(eij∗=1)q2j=∑i=1nsI(eij∗=−1).
 MathType@MTEF@5@5@+=feaafiart1ev1aaatCvAUfKttLearuWrP9MDH5MBPbIqV92AaeXatLxBI9gBaebbnrfifHhDYfgasaacH8akY=wiFfYdH8Gipec8Eeeu0xXdbba9frFj0=OqFfea0dXdd9vqai=hGuQ8kuc9pgc9s8qqaq=dirpe0xb9q8qiLsFr0=vr0=vr0dc8meaabaqaciaacaGaaeqabaqabeGadaaakeaafaqabeqacaaabaGaemyCae3aaSbaaSqaaiabigdaXiabdQgaQbqabaGccqGH9aqpdaaeWbqaaiabdMeajnaabmaabaGaemyzau2aa0baaSqaaiabdMgaPjabdQgaQbqaaiabgEHiQaaakiabg2da9iabigdaXaGaayjkaiaawMcaaaWcbaGaemyAaKMaeyypa0JaeGymaedabaGaemOBa42aaSbaaWqaaiabdohaZbqabaaaniabggHiLdaakeaacqWGXbqCdaWgaaWcbaGaeGOmaiJaemOAaOgabeaakiabg2da9maaqahabaGaemysaK0aaeWaaeaacqWGLbqzdaqhaaWcbaGaemyAaKMaemOAaOgabaGaey4fIOcaaOGaeyypa0JaeyOeI0IaeGymaedacaGLOaGaayzkaaaaleaacqWGPbqAcqGH9aqpcqaIXaqmaeaacqWGUbGBdaWgaaadbaGaem4Camhabeaaa0GaeyyeIuoakiabc6caUaaaaaa@5D46@

For each of the *g *groups of interest, each array *j *was then splitted in two disjoint sets of genes: one with exactly the *n*_*g *_elements of the group, and other constituted by the remaining *n*_*s*_-*n*_*g *_genes. One should also consider two random variables Y_1*j *_^(*g*) ^and Y_2*j *_^(*g*) ^representing the number of induced genes in the first and second group, respectively.

Under the null hypothesis of no biological activity for *g *in sample *j*, Segal et al [22] propose that Y_1*j *_^(*g*)^~hypergeometric(*n*_*g*_; *n*_*s*_-*n*_*g*_; *q*_1*j*_) and Y_2*j *_^(*g*)^~hypergeometric(*n*_*g*_; *n*_*s*_-*n*_*g*_; *q*_2*j*_). Intuitively, the absence of biological activity makes the probability of an induced (or repressed) gene to belong to the first group equal to 1/2 and *p*-values associated with the observed values y_1*j *_^(*g*) ^and y_2*j *_^(*g*) ^can be easily calculated. This procedure defined a new matrix *P*_*gj *_where the element *p*_*gj *_represents the fraction of induced genes of group *g *in the array *j*, or zero for the non-significant modules (we assumed a significance level *α *= 0.05). In the cases where the significance is for the repressed genes, we just made the fraction *p*_*gj *_negative. The arrays were then categorized in biological types t = 1,, *t *and for each pair of gene set *g *and biological type *t*, we count the number of arrays of type *t *that showed gene set *g *induced (repressed). We then used the hypergeometric distribution to classify each gene group as significantly induced or repressed across the biological types (p value < 0.05).

The biological types are defined as following: *n*_t _= 1 means that the macaque was not infected, *n*_t _= 2 means death 1 to 3 days after infection, *n*_t _= 3 means death 4 to 6 days after infection and *n*_t _= 4 means death after the 6^th ^day. *n*_t _= 2 was also labeled Early Death (ED) and *n*_t _= 3 and *n*_t _= 4 were labeled Late Death (LD). The contribution of molecules for activation of a significantly altered module was computed for time course and "ED and LD" analyses [see Additional files 1 and 2]. Using these values, it is possible to evaluate the influence of each gene in the module activation or repression (*p*-value).

### Relevance Networks (RNs)

For a better understanding of the Relevance Networks (RN) method, a definition of the term *interaction *is required. Here we are interested in a more general definition of protein interaction, which goes beyond physical interaction and includes also functional or phylogenetic relationships. Interaction here is inferred by significant correlation between two variables (i.e. molecules), which may (but not necessarily do) physically interact with each other. Aiming to identify such correlations, the Relevance Networks (RN) method, initially proposed by Butte et al [11], was employed. The method computes the squared linear Pearson correlation coefficient, rij2
 MathType@MTEF@5@5@+=feaafiart1ev1aaatCvAUfKttLearuWrP9MDH5MBPbIqV92AaeXatLxBI9gBaebbnrfifHhDYfgasaacH8akY=wiFfYdH8Gipec8Eeeu0xXdbba9frFj0=OqFfea0dXdd9vqai=hGuQ8kuc9pgc9s8qqaq=dirpe0xb9q8qiLsFr0=vr0=vr0dc8meaabaqaciaacaGaaeqabaqabeGadaaakeaabaGaemOCai3aa0baaSqaaiabdMgaPjabdQgaQbqaaiabbkdaYaaaaaaa@31EA@, between all gene pairs for each biological type (as described in the active modules section of methods), defining a fully connected graph. Using a re-sampling method, the algorithm estimates a cut-off value, *c*, to split the graph into small sub-graphs where rij2
 MathType@MTEF@5@5@+=feaafiart1ev1aaatCvAUfKttLearuWrP9MDH5MBPbIqV92AaeXatLxBI9gBaebbnrfifHhDYfgasaacH8akY=wiFfYdH8Gipec8Eeeu0xXdbba9frFj0=OqFfea0dXdd9vqai=hGuQ8kuc9pgc9s8qqaq=dirpe0xb9q8qiLsFr0=vr0=vr0dc8meaabaqaciaacaGaaeqabaqabeGadaaakeaabaGaemOCai3aa0baaSqaaiabdMgaPjabdQgaQbqaaiabbkdaYaaaaaaa@31EA@ > *c*. These sub-graphs are the relevance networks (RNs).

In order to identify significant differences between two RNs, we adapted this method to contrast two distinct conditions. Therefore, the linear Pearson correlation coefficients between every pair of genes in each condition, rij1
 MathType@MTEF@5@5@+=feaafiart1ev1aaatCvAUfKttLearuWrP9MDH5MBPbIqV92AaeXatLxBI9gBaebbnrfifHhDYfgasaacH8akY=wiFfYdH8Gipec8Eeeu0xXdbba9frFj0=OqFfea0dXdd9vqai=hGuQ8kuc9pgc9s8qqaq=dirpe0xb9q8qiLsFr0=vr0=vr0dc8meaabaqaciaacaGaaeqabaqabeGadaaakeaabaGaemOCai3aa0baaSqaaiabdMgaPjabdQgaQbqaaiabbgdaXaaaaaaa@31E8@ and rij2
 MathType@MTEF@5@5@+=feaafiart1ev1aaatCvAUfKttLearuWrP9MDH5MBPbIqV92AaeXatLxBI9gBaebbnrfifHhDYfgasaacH8akY=wiFfYdH8Gipec8Eeeu0xXdbba9frFj0=OqFfea0dXdd9vqai=hGuQ8kuc9pgc9s8qqaq=dirpe0xb9q8qiLsFr0=vr0=vr0dc8meaabaqaciaacaGaaeqabaqabeGadaaakeaabaGaemOCai3aa0baaSqaaiabdMgaPjabdQgaQbqaaiabbkdaYaaaaaaa@31EA@ are computed and then a Fisher's Z-transformation [23] is used to translate the probability distribution of the random variable rij1
 MathType@MTEF@5@5@+=feaafiart1ev1aaatCvAUfKttLearuWrP9MDH5MBPbIqV92AaeXatLxBI9gBaebbnrfifHhDYfgasaacH8akY=wiFfYdH8Gipec8Eeeu0xXdbba9frFj0=OqFfea0dXdd9vqai=hGuQ8kuc9pgc9s8qqaq=dirpe0xb9q8qiLsFr0=vr0=vr0dc8meaabaqaciaacaGaaeqabaqabeGadaaakeaabaGaemOCai3aa0baaSqaaiabdMgaPjabdQgaQbqaaiabbgdaXaaaaaaa@31E8@ - rij2
 MathType@MTEF@5@5@+=feaafiart1ev1aaatCvAUfKttLearuWrP9MDH5MBPbIqV92AaeXatLxBI9gBaebbnrfifHhDYfgasaacH8akY=wiFfYdH8Gipec8Eeeu0xXdbba9frFj0=OqFfea0dXdd9vqai=hGuQ8kuc9pgc9s8qqaq=dirpe0xb9q8qiLsFr0=vr0=vr0dc8meaabaqaciaacaGaaeqabaqabeGadaaakeaabaGaemOCai3aa0baaSqaaiabdMgaPjabdQgaQbqaaiabbkdaYaaaaaaa@31EA@ into an approximated standard normally distributed random variable, which permits the identification of pairs of genes that displayed significant (positive or negative) differences between rij1
 MathType@MTEF@5@5@+=feaafiart1ev1aaatCvAUfKttLearuWrP9MDH5MBPbIqV92AaeXatLxBI9gBaebbnrfifHhDYfgasaacH8akY=wiFfYdH8Gipec8Eeeu0xXdbba9frFj0=OqFfea0dXdd9vqai=hGuQ8kuc9pgc9s8qqaq=dirpe0xb9q8qiLsFr0=vr0=vr0dc8meaabaqaciaacaGaaeqabaqabeGadaaakeaabaGaemOCai3aa0baaSqaaiabdMgaPjabdQgaQbqaaiabbgdaXaaaaaaa@31E8@ and rij2
 MathType@MTEF@5@5@+=feaafiart1ev1aaatCvAUfKttLearuWrP9MDH5MBPbIqV92AaeXatLxBI9gBaebbnrfifHhDYfgasaacH8akY=wiFfYdH8Gipec8Eeeu0xXdbba9frFj0=OqFfea0dXdd9vqai=hGuQ8kuc9pgc9s8qqaq=dirpe0xb9q8qiLsFr0=vr0=vr0dc8meaabaqaciaacaGaaeqabaqabeGadaaakeaabaGaemOCai3aa0baaSqaaiabdMgaPjabdQgaQbqaaiabbkdaYaaaaaaa@31EA@ (*p *< 0.001). This type of analysis was accomplished for the 199 gene groups used in the active modules procedure, linking the findings with biological knowledge.

## Authors' contributions

All authors conceived the study. GHE implemented and adapted the methods. ACQS implemented the retrieval of the groups from KEGG. ACQS and TMV analyzed the results and drafted the latest version of the manuscript. ES, RAD, RO participated in the statistical modeling. All authors read and accepted the final version of this manuscript.

## Supplementary Material

Additional file 1 Contribution of each molecule for the time course active modules analysis. The contributions of individual molecules for each significantly altered module in the time course analysis. The contributions are represented by scores and the respective *p*-values. Click here for file

Additional file 2 Contribution of each molecule for the ED and LD active modules analysis. The contributions of individual molecules for each significantly altered module in the early and late death analysis. The contributions are represented by scores and the respective *p*-values. Click here for file

Additional file 3 Relevance networks for module ECM receptor + CAMs of day0 vs. day1–3 analysis. Edges from the graphs in first page (day 0) and second page (days 1–3) represent significant (p < 10^-3^) negative (green) or positive (red) correlation values. In the last graph (third page), instead of correlation, the edges represent the p-values of the significantly changes of the correlations. Click here for file

Additional file 4 Relevance networks for module ECM receptor + CAMs of day0 vs. day4–6 analysis. Edges from the graphs in first page (day 0) and second page (days 4–6) represent significant (p < 10^-3^) negative (green) or positive (red) correlation values. In the last graph (third page), instead of correlation, the edges represent the p-values of the significantly changes of the correlations. Click here for file

Additional file 5 Relevance networks for module Cytokines of day0 vs. day1–3. Edges from the graphs in first page (day 0) and second page (days 1–3) represent significant (p < 10^-3^) negative (green) or positive (red) correlation values. In the last graph (third page), instead of correlation, the edges represent the p-values of the significantly changes of the correlations. For this module the comparison of day 0 vs. days 4–6 has no statistically significant results. Click here for file

Additional file 6 Modules obtained from KEGG. We have selected 199 groups according to the level 4 of KEGG (updated on 04/17/2006). Click here for file
