# Supplementary material for: New insights about host response to smallpox using microarray data
Source: BMC Syst Biol. 2007 Aug 24;1:38. doi: 10.1186/1752-0509-1-38 (PMC2077868; doi:10.1186/1752-0509-1-38)
Supplement: Additional file 1 — Contribution of each molecule for the time course active modules analysis. The contributions of individual molecules for each significantly altered module in the time course analysis. The contributions are represented by scores and the respective p-values. [file 1752-0509-1-38-S1.html]

 R output 


import namespace="mml" implementation="#mathplayer"?


**Antigen\_processing\_and\_presentation**

| |  | Score | P.valor | | --- | --- | --- | | HLA-DMA | 3.7e+01 | 1.1e-15 | | HLA-DQB2 | 3.7e+01 | 4.6e-15 | | HLA-DRB3 | 3.5e+01 | 2.6e-13 | | HLA-DMB | 3.2e+01 | 5.4e-11 | | HLA-DQB1 | 2.8e+01 | 2.2e-08 | | HLA-DRA | 2.6e+01 | 1.3e-06 | | HLA-DPB1 | 2.5e+01 | 4.6e-06 | | HLA-DPA1 | 2.2e+01 | 1.1e-04 | | HLA-DQA2 | 2.0e+01 | 1.0e-03 | | CD74 | 1.8e+01 | 3.1e-03 | | HLA-DQA1 | 1.2e+01 | 1.9e-01 | | KLRC2 | 1.0e+01 | 3.1e-01 | | CTSL | 6.0e+00 | 7.4e-01 | | CTSB | 5.1e+00 | 8.2e-01 | | HLA-F | 3.0e+00 | 9.3e-01 | | CTSS | 2.6e+00 | 9.4e-01 | | PSME2 | 2.3e+00 | 9.5e-01 | | HSPA1A | 2.1e+00 | 9.6e-01 | | TAPBP | 2.1e+00 | 9.6e-01 | | TAP1 | 2.1e+00 | 9.6e-01 | | IFI30 | 2.1e+00 | 9.6e-01 | | CALR | 1.9e+00 | 9.6e-01 | | CD8A | 1.5e+00 | 9.7e-01 | | CD8B1 | 1.5e+00 | 9.7e-01 | | HLA-B | 1.2e+00 | 9.7e-01 | | PSME1 | 0.0e+00 | 9.9e-01 | | PSME3 | 0.0e+00 | 9.9e-01 | | HSPA5 | 0.0e+00 | 9.9e-01 | | HSPA8 | 0.0e+00 | 9.9e-01 | | HSPA9B | 0.0e+00 | 9.9e-01 | | HSPCA | 0.0e+00 | 9.9e-01 | | HLA-A | 0.0e+00 | 9.9e-01 | | HLA-C | 0.0e+00 | 9.9e-01 | | HLA-E | 0.0e+00 | 9.9e-01 | | HLA-G | 0.0e+00 | 9.9e-01 | | CANX | 0.0e+00 | 9.9e-01 | | B2M | 0.0e+00 | 9.9e-01 | | GRP58 | 0.0e+00 | 9.9e-01 | | HLA-DOA | 0.0e+00 | 9.9e-01 | | RFX5 | 0.0e+00 | 9.9e-01 | |

  

**Atrazine\_degradation**

| |  | Score | P.valor | | --- | --- | --- | | APOBEC3B | 9.4e+00 | 4.6e-05 | | APOBEC3G | 2.8e+00 | 8.2e-01 | | ADAR | 0.0e+00 | 1.0e-00 | |

  

**CAM\_ligands**

| |  | Score | P.valor | | --- | --- | --- | | THBS1 | 1.0e+01 | 4.1e-10 | | LAMA1 | 3.7e+00 | 4.3e-02 | | CD38 | 3.7e+00 | 4.3e-02 | | COL4A6 | 2.7e+00 | 1.5e-01 | | LAMB2 | 2.7e+00 | 1.5e-01 | | LAMB2 | 2.7e+00 | 1.5e-01 | | CD59 | 2.7e+00 | 1.5e-01 | | TGFB2 | 1.4e+00 | 4.2e-01 | | LCK | 1.4e+00 | 4.2e-01 | | FYN | 1.4e+00 | 4.2e-01 | | SEMA3C | 1.4e+00 | 4.2e-01 | | PTPRC | 1.4e+00 | 4.2e-01 | | COL4A1 | 0.0e+00 | 7.8e-01 | | COL4A2 | 0.0e+00 | 7.8e-01 | | LAMA3 | 0.0e+00 | 7.8e-01 | | FGA | 0.0e+00 | 7.8e-01 | | VEGFB | 0.0e+00 | 7.8e-01 | | MDK | 0.0e+00 | 7.8e-01 | | BST1 | 0.0e+00 | 7.8e-01 | | CYR61 | 0.0e+00 | 7.8e-01 | | LGALS1 | 0.0e+00 | 7.8e-01 | | LGALS8 | 0.0e+00 | 7.8e-01 | | ADAM9 | 0.0e+00 | 7.8e-01 | | ADAM17 | 0.0e+00 | 7.8e-01 | | SEMA3B | 0.0e+00 | 7.8e-01 | | SEMA4B | 0.0e+00 | 7.8e-01 | | SEMA4A | 0.0e+00 | 7.8e-01 | | LY6E | 0.0e+00 | 7.8e-01 | | PRG1 | 0.0e+00 | 7.8e-01 | |

  

**CD\_molecules**

| |  | Score | P.valor | | --- | --- | --- | | TLR4 | 3.6e+01 | 6.4e-23 | | CD163 | 3.3e+01 | 1.9e-18 | | CCR1 | 1.9e+01 | 2.9e-06 | | TLR2 | 1.9e+01 | 7.3e-06 | | ITGA2B | 1.7e+01 | 8.0e-05 | | MCAM | 1.6e+01 | 1.3e-04 | | SEMA4A | 1.6e+01 | 1.5e-04 | | CD38 | 1.6e+01 | 2.1e-04 | | IL6R | 1.6e+01 | 2.3e-04 | | CD5 | 1.6e+01 | 3.1e-04 | | CSF2RB | 1.5e+01 | 4.0e-04 | | IL2RB | 1.4e+01 | 1.4e-03 | | CD2 | 1.4e+01 | 1.6e-03 | | TNFSF10 | 1.4e+01 | 2.2e-03 | | PTPNS1 | 1.3e+01 | 3.8e-03 | | LILRB1 | 1.2e+01 | 1.3e-02 | | TLR8 | 1.2e+01 | 1.5e-02 | | CSF3R | 1.1e+01 | 2.3e-02 | | THY1 | 1.1e+01 | 2.3e-02 | | TLR1 | 1.0e+01 | 3.5e-02 | | LRP1 | 9.8e+00 | 5.4e-02 | | DAF | 9.4e+00 | 6.9e-02 | | SELP | 9.3e+00 | 7.2e-02 | | KLRC2 | 9.3e+00 | 7.4e-02 | | CSF2RA | 8.8e+00 | 9.6e-02 | | SELL | 8.2e+00 | 1.3e-01 | | CD9 | 8.2e+00 | 1.3e-01 | | CD68 | 8.1e+00 | 1.4e-01 | | CSF1R | 8.0e+00 | 1.4e-01 | | CD19 | 7.4e+00 | 1.9e-01 | | CD14 | 7.0e+00 | 2.2e-01 | | CXCR4 | 6.9e+00 | 2.3e-01 | | FCGR2B | 6.6e+00 | 2.7e-01 | | IL13RA1 | 6.6e+00 | 2.7e-01 | | LAIR1 | 6.3e+00 | 2.9e-01 | | CDW52 | 5.9e+00 | 3.4e-01 | | CD74 | 5.6e+00 | 3.8e-01 | | CD3D | 5.6e+00 | 3.8e-01 | | CD99 | 5.5e+00 | 3.9e-01 | | GGTL4 | 5.0e+00 | 4.4e-01 | | ENTPD1 | 5.0e+00 | 4.5e-01 | | IFITM2 | 4.6e+00 | 5.0e-01 | | IL2RG | 4.5e+00 | 5.1e-01 | | FCGR2A | 4.4e+00 | 5.3e-01 | | IFITM3 | 4.1e+00 | 5.6e-01 | | CD69 | 4.0e+00 | 5.7e-01 | | ICAM2 | 4.0e+00 | 5.7e-01 | | TNFSF8 | 4.0e+00 | 5.7e-01 | | ITGA5 | 3.9e+00 | 5.8e-01 | | PLXNC1 | 3.9e+00 | 5.8e-01 | | IL4R | 3.6e+00 | 6.2e-01 | | IGLL1 | 3.3e+00 | 6.6e-01 | | CD79A | 2.9e+00 | 6.9e-01 | | IL6ST | 2.9e+00 | 6.9e-01 | | SDC3 | 2.9e+00 | 6.9e-01 | | CD244 | 2.9e+00 | 6.9e-01 | | ITGA2 | 2.8e+00 | 7.1e-01 | | TNFRSF10D | 2.8e+00 | 7.1e-01 | | MS4A1 | 2.7e+00 | 7.2e-01 | | CD47 | 2.7e+00 | 7.2e-01 | | IL10RA | 2.7e+00 | 7.2e-01 | | ITGB1 | 2.6e+00 | 7.3e-01 | | BST1 | 2.3e+00 | 7.6e-01 | | ICAM3 | 2.3e+00 | 7.6e-01 | | IL2RA | 2.2e+00 | 7.6e-01 | | PECAM1 | 2.2e+00 | 7.6e-01 | | CD59 | 2.2e+00 | 7.6e-01 | | CD83 | 2.2e+00 | 7.6e-01 | | IL7R | 2.2e+00 | 7.6e-01 | | SELPLG | 2.2e+00 | 7.6e-01 | | BLR1 | 2.2e+00 | 7.6e-01 | | LILRB2 | 2.1e+00 | 7.8e-01 | | ADAM17 | 2.0e+00 | 7.8e-01 | | ITGAL | 1.8e+00 | 8.0e-01 | | PTPRC | 1.8e+00 | 8.0e-01 | | CD48 | 1.8e+00 | 8.0e-01 | | ITGA6 | 1.8e+00 | 8.0e-01 | | CD53 | 1.8e+00 | 8.0e-01 | | NT5C3 | 1.8e+00 | 8.0e-01 | | IL10RB | 1.8e+00 | 8.0e-01 | | TNFRSF13C | 1.8e+00 | 8.0e-01 | | BTN3A3 | 1.8e+00 | 8.0e-01 | | BTN3A1 | 1.8e+00 | 8.0e-01 | | CD3G | 0.0e+00 | 9.2e-01 | | CD8A | 0.0e+00 | 9.2e-01 | | CD8B1 | 0.0e+00 | 9.2e-01 | | FCGR3A | 0.0e+00 | 9.2e-01 | | ITGB2 | 0.0e+00 | 9.2e-01 | | FCER2 | 0.0e+00 | 9.2e-01 | | DPP4 | 0.0e+00 | 9.2e-01 | | DPP8 | 0.0e+00 | 9.2e-01 | | DPP9 | 0.0e+00 | 9.2e-01 | | TNFRSF7 | 0.0e+00 | 9.2e-01 | | CD37 | 0.0e+00 | 9.2e-01 | | CD44 | 0.0e+00 | 9.2e-01 | | ITGA3 | 0.0e+00 | 9.2e-01 | | ITGAV | 0.0e+00 | 9.2e-01 | | ICAM1 | 0.0e+00 | 9.2e-01 | | TNFSF7 | 0.0e+00 | 9.2e-01 | | CD72 | 0.0e+00 | 9.2e-01 | | NT5C2 | 0.0e+00 | 9.2e-01 | | CD79B | 0.0e+00 | 9.2e-01 | | CD81 | 0.0e+00 | 9.2e-01 | | KAI1 | 0.0e+00 | 9.2e-01 | | LILRB4 | 0.0e+00 | 9.2e-01 | | LILRB3 | 0.0e+00 | 9.2e-01 | | CDW92 | 0.0e+00 | 9.2e-01 | | SLC3A2 | 0.0e+00 | 9.2e-01 | | SEMA4B | 0.0e+00 | 9.2e-01 | | ITGAE | 0.0e+00 | 9.2e-01 | | ENG | 0.0e+00 | 9.2e-01 | | TNFRSF1B | 0.0e+00 | 9.2e-01 | | CDH5 | 0.0e+00 | 9.2e-01 | | BSG | 0.0e+00 | 9.2e-01 | | ADAM8 | 0.0e+00 | 9.2e-01 | | ADAM10 | 0.0e+00 | 9.2e-01 | | CD164 | 0.0e+00 | 9.2e-01 | | ALCAM | 0.0e+00 | 9.2e-01 | | DDR1 | 0.0e+00 | 9.2e-01 | | IL17R | 0.0e+00 | 9.2e-01 | | IGF2R | 0.0e+00 | 9.2e-01 | | IFITM1 | 0.0e+00 | 9.2e-01 | | PRNP | 0.0e+00 | 9.2e-01 | | SLC4A1 | 0.0e+00 | 9.2e-01 | | GYPC | 0.0e+00 | 9.2e-01 | | BTN2A2 | 0.0e+00 | 9.2e-01 | | TLR5 | 0.0e+00 | 9.2e-01 | | CD97 | 0.0e+00 | 9.2e-01 | | KLRK1 | 0.0e+00 | 9.2e-01 | | BST2 | 0.0e+00 | 9.2e-01 | | NCR3 | 0.0e+00 | 9.2e-01 | |

  

**Cell\_adhesion\_molecules\_CAMs**

| |  | Score | P.valor | | --- | --- | --- | | HLA-DQB2 | 4.3e+01 | 2.7e-19 | | HLA-DMA | 3.9e+01 | 2.0e-15 | | HLA-DRB3 | 3.6e+01 | 7.7e-13 | | HLA-DMB | 3.4e+01 | 2.7e-11 | | HLA-DQB1 | 3.2e+01 | 7.3e-10 | | HLA-DRA | 2.7e+01 | 1.4e-06 | | HLA-DPA1 | 2.6e+01 | 2.4e-06 | | HLA-DPB1 | 2.6e+01 | 5.2e-06 | | HLA-DQA2 | 2.4e+01 | 2.2e-05 | | PLXNB2 | 2.0e+01 | 1.9e-03 | | ITGA2B | 2.0e+01 | 2.2e-03 | | MCAM | 1.6e+01 | 3.5e-02 | | SELP | 1.3e+01 | 1.2e-01 | | HLA-DQA1 | 1.3e+01 | 1.4e-01 | | CD68 | 1.2e+01 | 1.7e-01 | | CD2 | 1.2e+01 | 1.9e-01 | | ITGB5 | 1.1e+01 | 2.9e-01 | | CSPG2 | 9.3e+00 | 4.2e-01 | | CD99 | 9.1e+00 | 4.4e-01 | | SDC3 | 8.0e+00 | 5.5e-01 | | ITGB1 | 7.9e+00 | 5.6e-01 | | ITGA2 | 6.2e+00 | 7.2e-01 | | ITGB7 | 5.9e+00 | 7.4e-01 | | THY1 | 4.3e+00 | 8.6e-01 | | ITGAL | 4.0e+00 | 8.7e-01 | | ITGA5 | 3.7e+00 | 8.9e-01 | | PTPRC | 3.7e+00 | 8.9e-01 | | ICAM2 | 3.2e+00 | 9.1e-01 | | SELL | 2.3e+00 | 9.4e-01 | | ESAM | 1.8e+00 | 9.5e-01 | | HLA-B | 1.6e+00 | 9.6e-01 | | CD8A | 1.6e+00 | 9.6e-01 | | CD8B1 | 1.6e+00 | 9.6e-01 | | CD48 | 1.6e+00 | 9.6e-01 | | ITGA6 | 1.6e+00 | 9.6e-01 | | GLG1 | 1.6e+00 | 9.6e-01 | | ICAM1 | 0.0e+00 | 9.8e-01 | | ICAM3 | 0.0e+00 | 9.8e-01 | | HLA-A | 0.0e+00 | 9.8e-01 | | HLA-C | 0.0e+00 | 9.8e-01 | | HLA-E | 0.0e+00 | 9.8e-01 | | HLA-F | 0.0e+00 | 9.8e-01 | | HLA-G | 0.0e+00 | 9.8e-01 | | HLA-DOA | 0.0e+00 | 9.8e-01 | | ALCAM | 0.0e+00 | 9.8e-01 | | PECAM1 | 0.0e+00 | 9.8e-01 | | MADCAM1 | 0.0e+00 | 9.8e-01 | | ITGA3 | 0.0e+00 | 9.8e-01 | | ITGAV | 0.0e+00 | 9.8e-01 | | ITGAE | 0.0e+00 | 9.8e-01 | | ITGB2 | 0.0e+00 | 9.8e-01 | | CD44 | 0.0e+00 | 9.8e-01 | | CDH5 | 0.0e+00 | 9.8e-01 | | SELPLG | 0.0e+00 | 9.8e-01 | | CD164 | 0.0e+00 | 9.8e-01 | | PLXNC1 | 0.0e+00 | 9.8e-01 | | NLGN1 | 0.0e+00 | 9.8e-01 | |

  

**Cell\_cycle**

| |  | Score | P.valor | | --- | --- | --- | | BRRN1 | 1.2e+01 | 5.7e-14 | | PCNA | 9.8e+00 | 4.3e-09 | | MCM4 | 9.8e+00 | 4.3e-09 | | MCM3 | 9.6e+00 | 1.3e-08 | | MCM5 | 4.5e+00 | 1.3e-02 | | SMAD2 | 2.5e+00 | 1.6e-01 | | BUB1B | 2.5e+00 | 1.6e-01 | | YWHAQ | 2.1e+00 | 2.4e-01 | | CCND1 | 0.0e+00 | 7.5e-01 | | CCND2 | 0.0e+00 | 7.5e-01 | | CCND3 | 0.0e+00 | 7.5e-01 | | CDK4 | 0.0e+00 | 7.5e-01 | | RB1 | 0.0e+00 | 7.5e-01 | | RBL2 | 0.0e+00 | 7.5e-01 | | ABL1 | 0.0e+00 | 7.5e-01 | | HDAC1 | 0.0e+00 | 7.5e-01 | | E2F4 | 0.0e+00 | 7.5e-01 | | GSK3B | 0.0e+00 | 7.5e-01 | | TGFB2 | 0.0e+00 | 7.5e-01 | | SMAD3 | 0.0e+00 | 7.5e-01 | | SMAD4 | 0.0e+00 | 7.5e-01 | | CDKN2A | 0.0e+00 | 7.5e-01 | | CDKN2D | 0.0e+00 | 7.5e-01 | | CDKN1B | 0.0e+00 | 7.5e-01 | | CDKN1A | 0.0e+00 | 7.5e-01 | | CCNE2 | 0.0e+00 | 7.5e-01 | | CDK2 | 0.0e+00 | 7.5e-01 | | SKP1A | 0.0e+00 | 7.5e-01 | | CCNB1 | 0.0e+00 | 7.5e-01 | | CDC25B | 0.0e+00 | 7.5e-01 | | YWHAB | 0.0e+00 | 7.5e-01 | | YWHAE | 0.0e+00 | 7.5e-01 | | WEE1 | 0.0e+00 | 7.5e-01 | | CCNH | 0.0e+00 | 7.5e-01 | | CDC27 | 0.0e+00 | 7.5e-01 | | ANAPC7 | 0.0e+00 | 7.5e-01 | | BUB3 | 0.0e+00 | 7.5e-01 | | MAD1L1 | 0.0e+00 | 7.5e-01 | | ATM | 0.0e+00 | 7.5e-01 | | TP53 | 0.0e+00 | 7.5e-01 | | CREBBP | 0.0e+00 | 7.5e-01 | | EP300 | 0.0e+00 | 7.5e-01 | | PRKDC | 0.0e+00 | 7.5e-01 | | ORC2L | 0.0e+00 | 7.5e-01 | | ORC4L | 0.0e+00 | 7.5e-01 | | MCM2 | 0.0e+00 | 7.5e-01 | | MCM7 | 0.0e+00 | 7.5e-01 | | FBXW7 | 0.0e+00 | 7.5e-01 | | CKS1B | 0.0e+00 | 7.5e-01 | | CSPG6 | 0.0e+00 | 7.5e-01 | | RAD21 | 0.0e+00 | 7.5e-01 | |

  

**Complement\_and\_coagulation\_cascades**

| |  | Score | P.valor | | --- | --- | --- | | DAF | 1.5e+01 | 1.5e-07 | | C3AR1 | 1.4e+01 | 3.9e-07 | | C2 | 3.3e+00 | 4.9e-01 | | CD59 | 3.3e+00 | 4.9e-01 | | F2R | 1.9e+00 | 7.4e-01 | | C1QB | 1.9e+00 | 7.4e-01 | | MBL2 | 1.9e+00 | 7.4e-01 | | DF | 1.5e+00 | 7.9e-01 | | F5 | 0.0e+00 | 9.3e-01 | | FGA | 0.0e+00 | 9.3e-01 | | SERPINF2 | 0.0e+00 | 9.3e-01 | | C4A | 0.0e+00 | 9.3e-01 | | C8A | 0.0e+00 | 9.3e-01 | |

  

**Cytokinecytokine\_receptor\_interaction**

| |  | Score | P.valor | | --- | --- | --- | | CX3CR1 | 1.8e+01 | 1.3e-06 | | PPBP | 1.7e+01 | 7.5e-06 | | PF4 | 1.4e+01 | 3.9e-04 | | CSF3R | 1.4e+01 | 4.9e-04 | | TGFB2 | 1.2e+01 | 4.4e-03 | | CSF2RB | 1.2e+01 | 5.3e-03 | | CCL4 | 1.1e+01 | 1.2e-02 | | CCR1 | 1.0e+01 | 1.8e-02 | | IL6R | 1.0e+01 | 2.1e-02 | | IL8 | 1.0e+01 | 2.8e-02 | | CSF1R | 9.7e+00 | 3.5e-02 | | CXCR4 | 8.8e+00 | 6.4e-02 | | CSF2RA | 7.6e+00 | 1.3e-01 | | IL2RG | 7.6e+00 | 1.3e-01 | | IL4R | 6.9e+00 | 1.9e-01 | | IL7R | 6.8e+00 | 2.0e-01 | | IL13RA1 | 6.0e+00 | 2.8e-01 | | CCL3 | 5.9e+00 | 2.9e-01 | | IL10RA | 5.5e+00 | 3.5e-01 | | TNFSF8 | 3.8e+00 | 5.7e-01 | | IFNGR2 | 3.2e+00 | 6.4e-01 | | IL2RB | 2.9e+00 | 6.9e-01 | | IL11 | 2.4e+00 | 7.4e-01 | | IL11RA | 2.4e+00 | 7.4e-01 | | FLT3LG | 2.2e+00 | 7.6e-01 | | IL24 | 2.2e+00 | 7.6e-01 | | TNFSF10 | 2.2e+00 | 7.6e-01 | | IL2RA | 2.2e+00 | 7.6e-01 | | TNFRSF14 | 2.2e+00 | 7.6e-01 | | TNFRSF13C | 1.9e+00 | 7.9e-01 | | TGFBR2 | 1.9e+00 | 7.9e-01 | | CXCL13 | 1.7e+00 | 8.1e-01 | | TGFBR1 | 1.7e+00 | 8.1e-01 | | BLR1 | 1.6e+00 | 8.2e-01 | | CCL5 | 0.0e+00 | 9.3e-01 | | CCL21 | 0.0e+00 | 9.3e-01 | | CCL8 | 0.0e+00 | 9.3e-01 | | PRL | 0.0e+00 | 9.3e-01 | | VEGFB | 0.0e+00 | 9.3e-01 | | IL10 | 0.0e+00 | 9.3e-01 | | TNFSF7 | 0.0e+00 | 9.3e-01 | | INHBB | 0.0e+00 | 9.3e-01 | | BMP8A | 0.0e+00 | 9.3e-01 | | IL6ST | 0.0e+00 | 9.3e-01 | | IL15RA | 0.0e+00 | 9.3e-01 | | IL10RB | 0.0e+00 | 9.3e-01 | | TNFRSF10D | 0.0e+00 | 9.3e-01 | | TNFRSF21 | 0.0e+00 | 9.3e-01 | | TNFRSF1B | 0.0e+00 | 9.3e-01 | | TNFRSF7 | 0.0e+00 | 9.3e-01 | | ACVR1 | 0.0e+00 | 9.3e-01 | | BMPR2 | 0.0e+00 | 9.3e-01 | | IL17R | 0.0e+00 | 9.3e-01 | |

  

**Cytokines**

| |  | Score | P.valor | | --- | --- | --- | | PPBP | 1.9e+01 | 4.6e-11 | | PF4 | 1.2e+01 | 1.1e-04 | | CCL4 | 9.7e+00 | 4.8e-03 | | TGFB2 | 8.2e+00 | 2.5e-02 | | CCL3 | 7.6e+00 | 4.2e-02 | | IL8 | 7.6e+00 | 4.2e-02 | | GDF1 | 6.5e+00 | 1.0e-01 | | IL24 | 4.2e+00 | 3.6e-01 | | TNFSF8 | 3.7e+00 | 4.5e-01 | | CCL5 | 2.5e+00 | 6.3e-01 | | FLT3LG | 2.1e+00 | 6.9e-01 | | IL11 | 0.0e+00 | 9.1e-01 | | PRL | 0.0e+00 | 9.1e-01 | | IL10 | 0.0e+00 | 9.1e-01 | | VEGFB | 0.0e+00 | 9.1e-01 | | EFNA1 | 0.0e+00 | 9.1e-01 | | EFNA5 | 0.0e+00 | 9.1e-01 | | GAS6 | 0.0e+00 | 9.1e-01 | | TNFSF7 | 0.0e+00 | 9.1e-01 | | TNFSF10 | 0.0e+00 | 9.1e-01 | | BMP8A | 0.0e+00 | 9.1e-01 | | INHBB | 0.0e+00 | 9.1e-01 | | CCL8 | 0.0e+00 | 9.1e-01 | | CCL21 | 0.0e+00 | 9.1e-01 | | CXCL13 | 0.0e+00 | 9.1e-01 | |

  

**Folate\_biosynthesis**

| |  | Score | P.valor | | --- | --- | --- | | LGP2 | 1.2e+01 | 1.0e-08 | | ALPL | 8.5e+00 | 1.3e-04 | | SMARCA5 | 1.7e+00 | 6.1e-01 | | GGH | 1.7e+00 | 6.1e-01 | | ALPI | 0.0e+00 | 8.9e-01 | | ERCC3 | 0.0e+00 | 8.9e-01 | | DDX47 | 0.0e+00 | 8.9e-01 | | DDX54 | 0.0e+00 | 8.9e-01 | | FBXO18 | 0.0e+00 | 8.9e-01 | | DDX18 | 0.0e+00 | 8.9e-01 | | DDX23 | 0.0e+00 | 8.9e-01 | |

  

**Galactose\_metabolism**

| |  | Score | P.valor | | --- | --- | --- | | MGAM | 8.0e+00 | 3.9e-06 | | HK3 | 6.4e+00 | 4.0e-04 | | UGP2 | 1.6e+00 | 5.0e-01 | | GALE | 0.0e+00 | 8.7e-01 | | PGM1 | 0.0e+00 | 8.7e-01 | | HK1 | 0.0e+00 | 8.7e-01 | | GLA | 0.0e+00 | 8.7e-01 | | PFKL | 0.0e+00 | 8.7e-01 | | PFKM | 0.0e+00 | 8.7e-01 | | RDH11 | 0.0e+00 | 8.7e-01 | |

  

**gammaHexachlorocyclohexane\_degradation**

| |  | Score | P.valor | | --- | --- | --- | | ALPL | 1.2e+01 | 7.3e-06 | | DHRS7 | 9.7e+00 | 1.5e-03 | | ACP5 | 4.2e+00 | 4.2e-01 | | DHRS3 | 2.1e+00 | 7.9e-01 | | ACP1 | 1.4e+00 | 8.8e-01 | | CYP3A4 | 0.0e+00 | 9.7e-01 | | ACP6 | 0.0e+00 | 9.7e-01 | | ALPI | 0.0e+00 | 9.7e-01 | |

  

**Glycolysis**

| |  | Score | P.valor | | --- | --- | --- | | BPGM | 7.5e+00 | 2.9e-09 | | BPGM | 7.5e+00 | 2.9e-09 | | BPGM | 7.5e+00 | 2.9e-09 | | ALDOA | 2.6e+00 | 4.4e-02 | | LDHA | 2.6e+00 | 4.4e-02 | | ENO2 | 2.2e+00 | 8.6e-02 | | PGM1 | 2.2e+00 | 8.6e-02 | | HK1 | 0.0e+00 | 7.0e-01 | | HK3 | 0.0e+00 | 7.0e-01 | | PFKL | 0.0e+00 | 7.0e-01 | | PFKM | 0.0e+00 | 7.0e-01 | | GAPD | 0.0e+00 | 7.0e-01 | | PGK1 | 0.0e+00 | 7.0e-01 | | PGAM1 | 0.0e+00 | 7.0e-01 | | ENO1 | 0.0e+00 | 7.0e-01 | | PKM2 | 0.0e+00 | 7.0e-01 | | PDHA1 | 0.0e+00 | 7.0e-01 | | PDHA2 | 0.0e+00 | 7.0e-01 | | PDHB | 0.0e+00 | 7.0e-01 | | DLAT | 0.0e+00 | 7.0e-01 | | DLD | 0.0e+00 | 7.0e-01 | | LDHB | 0.0e+00 | 7.0e-01 | | ADH5 | 0.0e+00 | 7.0e-01 | | ALDH9A1 | 0.0e+00 | 7.0e-01 | | ALDH3A2 | 0.0e+00 | 7.0e-01 | | ACAS2L | 0.0e+00 | 7.0e-01 | | PGAM1 | 0.0e+00 | 7.0e-01 | | PGAM1 | 0.0e+00 | 7.0e-01 | |

  

**GTPbinding\_proteins**

| |  | Score | P.valor | | --- | --- | --- | | RAB31 | 1.0e+01 | 2.5e-09 | | RHOB | 9.3e+00 | 3.4e-08 | | ARL4A | 5.0e+00 | 5.4e-03 | | GNB1 | 4.9e+00 | 6.6e-03 | | RAB10 | 4.7e+00 | 9.1e-03 | | GNA15 | 4.6e+00 | 9.8e-03 | | RAP2A | 2.8e+00 | 1.3e-01 | | GNG10 | 2.3e+00 | 2.1e-01 | | RHOG | 2.3e+00 | 2.1e-01 | | RAB1A | 2.3e+00 | 2.1e-01 | | GNAI3 | 2.1e+00 | 2.4e-01 | | RHOQ | 2.1e+00 | 2.4e-01 | | RAB27A | 2.1e+00 | 2.4e-01 | | GNAI2 | 0.0e+00 | 7.6e-01 | | GNAT1 | 0.0e+00 | 7.6e-01 | | GNA12 | 0.0e+00 | 7.6e-01 | | GNB2 | 0.0e+00 | 7.6e-01 | | GNG11 | 0.0e+00 | 7.6e-01 | | NRAS | 0.0e+00 | 7.6e-01 | | RRAS2 | 0.0e+00 | 7.6e-01 | | RALA | 0.0e+00 | 7.6e-01 | | RALB | 0.0e+00 | 7.6e-01 | | RAP1A | 0.0e+00 | 7.6e-01 | | RAP1B | 0.0e+00 | 7.6e-01 | | DIRAS2 | 0.0e+00 | 7.6e-01 | | RHOA | 0.0e+00 | 7.6e-01 | | RHOC | 0.0e+00 | 7.6e-01 | | RAC1 | 0.0e+00 | 7.6e-01 | | RAC2 | 0.0e+00 | 7.6e-01 | | CDC42 | 0.0e+00 | 7.6e-01 | | RHOT1 | 0.0e+00 | 7.6e-01 | | RHOT2 | 0.0e+00 | 7.6e-01 | | RHOH | 0.0e+00 | 7.6e-01 | | RAB1B | 0.0e+00 | 7.6e-01 | | RAB2 | 0.0e+00 | 7.6e-01 | | RAB14 | 0.0e+00 | 7.6e-01 | | RAB5A | 0.0e+00 | 7.6e-01 | | RAB5B | 0.0e+00 | 7.6e-01 | | RAB5C | 0.0e+00 | 7.6e-01 | | RAB21 | 0.0e+00 | 7.6e-01 | | RAB22A | 0.0e+00 | 7.6e-01 | | RAB6A | 0.0e+00 | 7.6e-01 | | RAB7 | 0.0e+00 | 7.6e-01 | | RAB8A | 0.0e+00 | 7.6e-01 | | RAB24 | 0.0e+00 | 7.6e-01 | | RAB43 | 0.0e+00 | 7.6e-01 | | RAN | 0.0e+00 | 7.6e-01 | | SARA2 | 0.0e+00 | 7.6e-01 | | ARL10B | 0.0e+00 | 7.6e-01 | | ARL10C | 0.0e+00 | 7.6e-01 | | TRIM23 | 0.0e+00 | 7.6e-01 | |

  

**Hematopoietic\_cell\_lineage**

| |  | Score | P.valor | | --- | --- | --- | | HLA-DRB3 | 3.1e+01 | 3.0e-13 | | HLA-DRA | 2.5e+01 | 1.0e-07 | | ITGA2B | 1.3e+01 | 4.6e-02 | | CD19 | 1.1e+01 | 8.3e-02 | | CD38 | 1.1e+01 | 9.3e-02 | | IL11RA | 9.9e+00 | 1.8e-01 | | CD9 | 9.9e+00 | 1.8e-01 | | CD3D | 9.8e+00 | 1.9e-01 | | CD2 | 8.7e+00 | 2.8e-01 | | IL7R | 8.1e+00 | 3.4e-01 | | FLT3LG | 7.9e+00 | 3.7e-01 | | CSF1R | 7.4e+00 | 4.2e-01 | | MS4A1 | 6.9e+00 | 4.8e-01 | | IL6R | 6.4e+00 | 5.4e-01 | | CD5 | 6.3e+00 | 5.4e-01 | | CSF2RA | 5.9e+00 | 6.0e-01 | | CSF3R | 5.9e+00 | 6.0e-01 | | IL4R | 5.7e+00 | 6.2e-01 | | CD14 | 5.7e+00 | 6.2e-01 | | CD8A | 3.7e+00 | 8.1e-01 | | ITGA6 | 3.6e+00 | 8.2e-01 | | ITGA5 | 3.3e+00 | 8.4e-01 | | IL11 | 2.4e+00 | 9.0e-01 | | IL2RA | 2.1e+00 | 9.1e-01 | | CD37 | 2.1e+00 | 9.1e-01 | | ITGA2 | 2.1e+00 | 9.1e-01 | | DAF | 1.9e+00 | 9.2e-01 | | CD59 | 1.9e+00 | 9.2e-01 | | CD8B1 | 1.6e+00 | 9.3e-01 | | CD3G | 1.4e+00 | 9.4e-01 | | CD44 | 0.0e+00 | 9.7e-01 | | FCER2 | 0.0e+00 | 9.7e-01 | | ITGA3 | 0.0e+00 | 9.7e-01 | |

  

**JakSTAT\_signaling\_pathway**

| |  | Score | P.valor | | --- | --- | --- | | CSF2RA | 1.2e+01 | 7.8e-07 | | IL2RA | 1.1e+01 | 5.3e-06 | | IL7R | 1.0e+01 | 1.8e-05 | | IFNGR2 | 9.0e+00 | 2.5e-04 | | CSF2RB | 8.7e+00 | 3.7e-04 | | CSF3R | 8.7e+00 | 3.7e-04 | | IL13RA1 | 6.7e+00 | 9.1e-03 | | SOCS1 | 5.8e+00 | 2.6e-02 | | CBLB | 5.5e+00 | 3.6e-02 | | IL6R | 4.3e+00 | 1.2e-01 | | IL4R | 2.9e+00 | 3.0e-01 | | SPRY1 | 2.6e+00 | 3.6e-01 | | CCND2 | 2.4e+00 | 4.0e-01 | | IL2RG | 2.4e+00 | 4.0e-01 | | IL11RA | 2.4e+00 | 4.0e-01 | | IL11 | 2.4e+00 | 4.0e-01 | | STAT5B | 2.1e+00 | 4.6e-01 | | CISH | 2.1e+00 | 4.6e-01 | | PIK3R3 | 2.1e+00 | 4.6e-01 | | JAK1 | 0.0e+00 | 8.2e-01 | | TYK2 | 0.0e+00 | 8.2e-01 | | STAT1 | 0.0e+00 | 8.2e-01 | | STAT3 | 0.0e+00 | 8.2e-01 | | STAT4 | 0.0e+00 | 8.2e-01 | | STAT5A | 0.0e+00 | 8.2e-01 | | STAT6 | 0.0e+00 | 8.2e-01 | | ISGF3G | 0.0e+00 | 8.2e-01 | | CREBBP | 0.0e+00 | 8.2e-01 | | EP300 | 0.0e+00 | 8.2e-01 | | SOCS3 | 0.0e+00 | 8.2e-01 | | PIM1 | 0.0e+00 | 8.2e-01 | | MYC | 0.0e+00 | 8.2e-01 | | CCND1 | 0.0e+00 | 8.2e-01 | | CCND3 | 0.0e+00 | 8.2e-01 | | BCL2L1 | 0.0e+00 | 8.2e-01 | | SPRY2 | 0.0e+00 | 8.2e-01 | | STAM2 | 0.0e+00 | 8.2e-01 | | PIAS3 | 0.0e+00 | 8.2e-01 | | PIAS1 | 0.0e+00 | 8.2e-01 | | GRB2 | 0.0e+00 | 8.2e-01 | | SOS1 | 0.0e+00 | 8.2e-01 | | PIK3CA | 0.0e+00 | 8.2e-01 | | PIK3CG | 0.0e+00 | 8.2e-01 | | PIK3R1 | 0.0e+00 | 8.2e-01 | | AKT2 | 0.0e+00 | 8.2e-01 | | PTPN11 | 0.0e+00 | 8.2e-01 | | PTPN6 | 0.0e+00 | 8.2e-01 | | IL2RB | 0.0e+00 | 8.2e-01 | | IL6ST | 0.0e+00 | 8.2e-01 | | IL10RA | 0.0e+00 | 8.2e-01 | | IL10RB | 0.0e+00 | 8.2e-01 | | IL15RA | 0.0e+00 | 8.2e-01 | | IL24 | 0.0e+00 | 8.2e-01 | | IL10 | 0.0e+00 | 8.2e-01 | | PRL | 0.0e+00 | 8.2e-01 | |

  

**Leukocyte\_transendothelial\_migration**

| |  | Score | P.valor | | --- | --- | --- | | MMP9 | 1.5e+01 | 7.8e-11 | | PPBP | 1.2e+01 | 2.5e-07 | | NCF4 | 9.9e+00 | 4.6e-05 | | IL8 | 9.5e+00 | 1.0e-04 | | PF4 | 7.4e+00 | 3.7e-03 | | THY1 | 7.2e+00 | 4.8e-03 | | CXCR4 | 5.1e+00 | 5.8e-02 | | ITGB1 | 5.0e+00 | 6.5e-02 | | CD99 | 5.0e+00 | 6.5e-02 | | PECAM1 | 2.9e+00 | 3.1e-01 | | ESAM | 2.4e+00 | 4.0e-01 | | PTK2 | 2.4e+00 | 4.0e-01 | | VCL | 2.4e+00 | 4.0e-01 | | VIL2 | 2.2e+00 | 4.4e-01 | | PIK3R3 | 2.2e+00 | 4.4e-01 | | CYBA | 2.2e+00 | 4.4e-01 | | MAPK14 | 2.2e+00 | 4.4e-01 | | PXN | 2.2e+00 | 4.4e-01 | | VASP | 2.2e+00 | 4.4e-01 | | CXCL13 | 2.2e+00 | 4.4e-01 | | ITGB2 | 0.0e+00 | 8.2e-01 | | ITGAL | 0.0e+00 | 8.2e-01 | | CDH5 | 0.0e+00 | 8.2e-01 | | MSN | 0.0e+00 | 8.2e-01 | | ACTB | 0.0e+00 | 8.2e-01 | | PIK3CA | 0.0e+00 | 8.2e-01 | | PIK3CG | 0.0e+00 | 8.2e-01 | | PIK3R1 | 0.0e+00 | 8.2e-01 | | RAC1 | 0.0e+00 | 8.2e-01 | | CYBB | 0.0e+00 | 8.2e-01 | | CTNNB1 | 0.0e+00 | 8.2e-01 | | CTNND1 | 0.0e+00 | 8.2e-01 | | CTNNA1 | 0.0e+00 | 8.2e-01 | | PTPN11 | 0.0e+00 | 8.2e-01 | | MAPK11 | 0.0e+00 | 8.2e-01 | | ICAM1 | 0.0e+00 | 8.2e-01 | | PLCG2 | 0.0e+00 | 8.2e-01 | | PRKCB1 | 0.0e+00 | 8.2e-01 | | GRLF1 | 0.0e+00 | 8.2e-01 | | RHOA | 0.0e+00 | 8.2e-01 | | MYL6 | 0.0e+00 | 8.2e-01 | | RAP1A | 0.0e+00 | 8.2e-01 | | RAP1B | 0.0e+00 | 8.2e-01 | | SIPA1 | 0.0e+00 | 8.2e-01 | | GNAI2 | 0.0e+00 | 8.2e-01 | | GNAI3 | 0.0e+00 | 8.2e-01 | | RASSF5 | 0.0e+00 | 8.2e-01 | | ITK | 0.0e+00 | 8.2e-01 | | VAV3 | 0.0e+00 | 8.2e-01 | | VAV1 | 0.0e+00 | 8.2e-01 | | RAC2 | 0.0e+00 | 8.2e-01 | | CDC42 | 0.0e+00 | 8.2e-01 | | RHOH | 0.0e+00 | 8.2e-01 | | ACTN1 | 0.0e+00 | 8.2e-01 | |

  

**Natural\_killer\_cell\_mediated\_cytotoxicity**

| |  | Score | P.valor | | --- | --- | --- | | KLRC2 | 1.9e+01 | 3.1e-14 | | PRF1 | 1.8e+01 | 4.6e-13 | | GZMB | 1.6e+01 | 1.3e-10 | | RRAS2 | 1.5e+01 | 2.9e-09 | | SH2D1A | 1.2e+01 | 1.4e-06 | | LCK | 6.9e+00 | 1.5e-02 | | CD244 | 5.0e+00 | 9.0e-02 | | NFATC2 | 4.6e+00 | 1.3e-01 | | ZAP70 | 4.2e+00 | 1.7e-01 | | LAT | 3.0e+00 | 3.3e-01 | | HLA-F | 2.7e+00 | 3.8e-01 | | ITGAL | 2.7e+00 | 3.8e-01 | | TYROBP | 2.7e+00 | 3.8e-01 | | PIK3CG | 2.7e+00 | 3.8e-01 | | TNFSF10 | 2.7e+00 | 3.8e-01 | | FCER1G | 2.5e+00 | 4.2e-01 | | FYN | 2.5e+00 | 4.2e-01 | | ICAM2 | 1.8e+00 | 5.4e-01 | | KLRK1 | 1.8e+00 | 5.4e-01 | | PPP3CC | 1.8e+00 | 5.4e-01 | | HLA-A | 0.0e+00 | 8.2e-01 | | HLA-B | 0.0e+00 | 8.2e-01 | | HLA-C | 0.0e+00 | 8.2e-01 | | HLA-E | 0.0e+00 | 8.2e-01 | | HLA-G | 0.0e+00 | 8.2e-01 | | PTPN6 | 0.0e+00 | 8.2e-01 | | PTPN11 | 0.0e+00 | 8.2e-01 | | ICAM1 | 0.0e+00 | 8.2e-01 | | ITGB2 | 0.0e+00 | 8.2e-01 | | VAV3 | 0.0e+00 | 8.2e-01 | | VAV1 | 0.0e+00 | 8.2e-01 | | RAC1 | 0.0e+00 | 8.2e-01 | | RAC2 | 0.0e+00 | 8.2e-01 | | MAP2K1 | 0.0e+00 | 8.2e-01 | | MAP2K2 | 0.0e+00 | 8.2e-01 | | MAPK1 | 0.0e+00 | 8.2e-01 | | FCGR3A | 0.0e+00 | 8.2e-01 | | NCR3 | 0.0e+00 | 8.2e-01 | | SYK | 0.0e+00 | 8.2e-01 | | LCP2 | 0.0e+00 | 8.2e-01 | | PLCG2 | 0.0e+00 | 8.2e-01 | | PIK3CA | 0.0e+00 | 8.2e-01 | | PIK3R1 | 0.0e+00 | 8.2e-01 | | PIK3R3 | 0.0e+00 | 8.2e-01 | | SHC1 | 0.0e+00 | 8.2e-01 | | GRB2 | 0.0e+00 | 8.2e-01 | | SOS1 | 0.0e+00 | 8.2e-01 | | NRAS | 0.0e+00 | 8.2e-01 | | RAF1 | 0.0e+00 | 8.2e-01 | | MICA | 0.0e+00 | 8.2e-01 | | CD48 | 0.0e+00 | 8.2e-01 | | PPP3CA | 0.0e+00 | 8.2e-01 | | PPP3CB | 0.0e+00 | 8.2e-01 | | CHP | 0.0e+00 | 8.2e-01 | | PPP3R1 | 0.0e+00 | 8.2e-01 | | NFATC1 | 0.0e+00 | 8.2e-01 | | NFATC3 | 0.0e+00 | 8.2e-01 | | PRKCB1 | 0.0e+00 | 8.2e-01 | | IFNGR2 | 0.0e+00 | 8.2e-01 | | TNFRSF10D | 0.0e+00 | 8.2e-01 | | CASP3 | 0.0e+00 | 8.2e-01 | | BID | 0.0e+00 | 8.2e-01 | |

  

**Neuroactive\_ligandreceptor\_interaction**

| |  | Score | P.valor | | --- | --- | --- | | FPR1 | 2.2e+01 | 3.4e-10 | | C3AR1 | 2.0e+01 | 4.4e-08 | | P2RY2 | 1.4e+01 | 7.4e-04 | | ADORA2B | 9.8e+00 | 4.6e-02 | | GZMA | 8.8e+00 | 9.3e-02 | | P2RY10 | 4.7e+00 | 5.6e-01 | | F2R | 3.9e+00 | 6.6e-01 | | PTAFR | 2.8e+00 | 7.9e-01 | | THRA | 2.8e+00 | 7.9e-01 | | PTGER4 | 2.4e+00 | 8.3e-01 | | ADRB2 | 2.3e+00 | 8.4e-01 | | EDG5 | 2.3e+00 | 8.4e-01 | | P2RX4 | 1.9e+00 | 8.7e-01 | | P2RY5 | 1.2e+00 | 9.2e-01 | | EDG1 | 1.2e+00 | 9.2e-01 | | EDG4 | 1.2e+00 | 9.2e-01 | | PTGER3 | 0.0e+00 | 9.7e-01 | | GABRB1 | 0.0e+00 | 9.7e-01 | | NR3C1 | 0.0e+00 | 9.7e-01 | | PRL | 0.0e+00 | 9.7e-01 | |

  

**Nitrogen\_metabolism**

| |  | Score | P.valor | | --- | --- | --- | | CA6 | 1.0e+01 | 1.1e-06 | | GLUD1 | 2.4e+00 | 4.6e-01 | | GLUL | 2.4e+00 | 4.6e-01 | | CA13 | 1.4e+00 | 6.9e-01 | | ASNS | 1.4e+00 | 6.9e-01 | | CA14 | 0.0e+00 | 9.1e-01 | | AMT | 0.0e+00 | 9.1e-01 | | CTH | 0.0e+00 | 9.1e-01 | |

  

**Parkinsons\_disease**

| |  | Score | P.valor | | --- | --- | --- | | UBE2L6 | 2.197 | 0.023 | | SNCA | 2.197 | 0.023 | | UBE1 | 0.000 | 0.841 | | UBE2G2 | 0.000 | 0.841 | | STX1A | 0.000 | 0.841 | | PARK7 | 0.000 | 0.841 | |

  

**Pores\_ion\_channels**

| |  | Score | P.valor | | --- | --- | --- | | HSPA1A | 6.9e+00 | 5.4e-05 | | HSPA5 | 0.0e+00 | 9.0e-01 | | HSPA8 | 0.0e+00 | 9.0e-01 | | HSPA9B | 0.0e+00 | 9.0e-01 | |

  

**Regulation\_of\_actin\_cytoskeleton**

| |  | Score | P.valor | | --- | --- | --- | | ITGA2B | 1.2e+01 | 9.6e-12 | | MYLK | 9.9e+00 | 1.3e-07 | | F2R | 9.6e+00 | 2.7e-07 | | ITGB5 | 9.6e+00 | 2.7e-07 | | VCL | 7.2e+00 | 2.0e-04 | | ITGA2 | 7.0e+00 | 2.8e-04 | | GSN | 7.0e+00 | 2.8e-04 | | ITGB1 | 5.3e+00 | 7.9e-03 | | CYFIP1 | 2.8e+00 | 1.7e-01 | | ITGAL | 2.6e+00 | 2.1e-01 | | PIK3CG | 2.6e+00 | 2.1e-01 | | CD14 | 2.4e+00 | 2.4e-01 | | PTK2 | 2.4e+00 | 2.4e-01 | | ARPC4 | 2.4e+00 | 2.4e-01 | | PIP5K1A | 2.3e+00 | 2.7e-01 | | ITGA3 | 0.0e+00 | 7.7e-01 | | ITGA5 | 0.0e+00 | 7.7e-01 | | ITGA6 | 0.0e+00 | 7.7e-01 | | ITGAV | 0.0e+00 | 7.7e-01 | | ITGAE | 0.0e+00 | 7.7e-01 | | ITGB2 | 0.0e+00 | 7.7e-01 | | ITGB7 | 0.0e+00 | 7.7e-01 | | GNA12 | 0.0e+00 | 7.7e-01 | | CRK | 0.0e+00 | 7.7e-01 | | CSK | 0.0e+00 | 7.7e-01 | | SOS1 | 0.0e+00 | 7.7e-01 | | NRAS | 0.0e+00 | 7.7e-01 | | RRAS2 | 0.0e+00 | 7.7e-01 | | ARHGEF6 | 0.0e+00 | 7.7e-01 | | PIK3CA | 0.0e+00 | 7.7e-01 | | PIK3R1 | 0.0e+00 | 7.7e-01 | | PIK3R3 | 0.0e+00 | 7.7e-01 | | VAV3 | 0.0e+00 | 7.7e-01 | | VAV1 | 0.0e+00 | 7.7e-01 | | TIAM1 | 0.0e+00 | 7.7e-01 | | RAF1 | 0.0e+00 | 7.7e-01 | | MAP2K1 | 0.0e+00 | 7.7e-01 | | MAP2K2 | 0.0e+00 | 7.7e-01 | | MAPK1 | 0.0e+00 | 7.7e-01 | | RHOA | 0.0e+00 | 7.7e-01 | | RAC1 | 0.0e+00 | 7.7e-01 | | RAC2 | 0.0e+00 | 7.7e-01 | | CDC42 | 0.0e+00 | 7.7e-01 | | PAK2 | 0.0e+00 | 7.7e-01 | | PPP1CA | 0.0e+00 | 7.7e-01 | | PPP1CB | 0.0e+00 | 7.7e-01 | | PPP1CC | 0.0e+00 | 7.7e-01 | | PPP1R12A | 0.0e+00 | 7.7e-01 | | SLC9A1 | 0.0e+00 | 7.7e-01 | | WAS | 0.0e+00 | 7.7e-01 | | WASL | 0.0e+00 | 7.7e-01 | | CYFIP2 | 0.0e+00 | 7.7e-01 | | HEM1 | 0.0e+00 | 7.7e-01 | | ARPC5 | 0.0e+00 | 7.7e-01 | | ARPC5L | 0.0e+00 | 7.7e-01 | | ARPC3 | 0.0e+00 | 7.7e-01 | | ARPC2 | 0.0e+00 | 7.7e-01 | | ACTB | 0.0e+00 | 7.7e-01 | | PXN | 0.0e+00 | 7.7e-01 | | VIL2 | 0.0e+00 | 7.7e-01 | | MSN | 0.0e+00 | 7.7e-01 | | TMSB4X | 0.0e+00 | 7.7e-01 | | TMSB4Y | 0.0e+00 | 7.7e-01 | | CFL2 | 0.0e+00 | 7.7e-01 | | IQGAP1 | 0.0e+00 | 7.7e-01 | | ACTN1 | 0.0e+00 | 7.7e-01 | | MYH9 | 0.0e+00 | 7.7e-01 | | ARHGEF1 | 0.0e+00 | 7.7e-01 | | GRLF1 | 0.0e+00 | 7.7e-01 | |

  

**Ribosome**

| |  | Score | P.valor | | --- | --- | --- | | RPS27 | 1.3e+01 | 9.8e-07 | | RPL35A | 1.1e+01 | 3.8e-05 | | RPS25 | 7.2e+00 | 2.0e-02 | | RPL28 | 6.7e+00 | 3.3e-02 | | RPS12 | 5.9e+00 | 6.8e-02 | | RPL35 | 5.2e+00 | 1.2e-01 | | RPL7A | 4.6e+00 | 1.8e-01 | | RPL5 | 3.9e+00 | 2.9e-01 | | RPL10 | 3.7e+00 | 3.1e-01 | | RPL13A | 3.7e+00 | 3.1e-01 | | RPL23A | 3.4e+00 | 3.5e-01 | | RPS14 | 3.2e+00 | 4.0e-01 | | RPL23 | 3.2e+00 | 4.0e-01 | | RPS10 | 2.9e+00 | 4.4e-01 | | RPS28 | 2.5e+00 | 5.3e-01 | | RPS4X | 2.2e+00 | 5.8e-01 | | RPL22 | 2.2e+00 | 5.8e-01 | | RPL11 | 1.9e+00 | 6.2e-01 | | RPS21 | 1.8e+00 | 6.5e-01 | | RPS13 | 9.9e-01 | 7.7e-01 | | RPL6 | 9.9e-01 | 7.7e-01 | | RPL36AL | 9.9e-01 | 7.7e-01 | | RPS5 | 0.0e+00 | 8.8e-01 | | RPS16 | 0.0e+00 | 8.8e-01 | | RPS23 | 0.0e+00 | 8.8e-01 | | RPS24 | 0.0e+00 | 8.8e-01 | | RPS29 | 0.0e+00 | 8.8e-01 | | LAMR1 | 0.0e+00 | 8.8e-01 | | RPL12 | 0.0e+00 | 8.8e-01 | | RPL15 | 0.0e+00 | 8.8e-01 | | RPL17 | 0.0e+00 | 8.8e-01 | | C15orf15 | 0.0e+00 | 8.8e-01 | | RPL27 | 0.0e+00 | 8.8e-01 | | RPL29 | 0.0e+00 | 8.8e-01 | | RPL32 | 0.0e+00 | 8.8e-01 | |

  

**Starch\_and\_sucrose\_metabolism**

| |  | Score | P.valor | | --- | --- | --- | | PYGL | 3.5e+01 | 9.8e-23 | | MGAM | 3.3e+01 | 9.7e-20 | | MGAM | 3.3e+01 | 9.7e-20 | | HK3 | 1.0e+01 | 5.0e-02 | | LGP2 | 9.2e+00 | 1.1e-01 | | AMY1A | 3.7e+00 | 7.2e-01 | | DDX54 | 2.2e+00 | 8.6e-01 | | SMARCA5 | 2.2e+00 | 8.6e-01 | | UGT1A10 | 1.8e+00 | 8.9e-01 | | UGP2 | 1.8e+00 | 8.9e-01 | | GUSB | 0.0e+00 | 9.7e-01 | | PGM1 | 0.0e+00 | 9.7e-01 | | HK1 | 0.0e+00 | 9.7e-01 | | ERCC3 | 0.0e+00 | 9.7e-01 | | DDX47 | 0.0e+00 | 9.7e-01 | | FBXO18 | 0.0e+00 | 9.7e-01 | | DDX18 | 0.0e+00 | 9.7e-01 | | DDX23 | 0.0e+00 | 9.7e-01 | |

  

**Streptomycin\_biosynthesis**

| |  | Score | P.valor | | --- | --- | --- | | IMPA2 | 4.8520 | 0.0023 | | HK3 | 3.4657 | 0.0786 | | HK1 | 0.0000 | 0.9831 | | PGM1 | 0.0000 | 0.9831 | |

  

**Type\_I\_diabetes\_mellitus**

| |  | Score | P.valor | | --- | --- | --- | | HLA-DMA | 2.6e+01 | 8.6e-06 | | HLA-DRB3 | 2.6e+01 | 1.1e-05 | | HLA-DQB2 | 2.5e+01 | 4.7e-05 | | HLA-DMB | 2.4e+01 | 9.5e-05 | | HLA-DQB1 | 2.3e+01 | 2.5e-04 | | HLA-DPB1 | 1.9e+01 | 1.0e-02 | | HLA-DRA | 1.7e+01 | 4.0e-02 | | HLA-DQA2 | 1.6e+01 | 9.0e-02 | | PRF1 | 1.6e+01 | 9.4e-02 | | HLA-DPA1 | 1.5e+01 | 1.3e-01 | | GZMB | 1.4e+01 | 2.1e-01 | | HLA-DQA1 | 7.2e+00 | 8.6e-01 | | HLA-F | 1.9e+00 | 9.9e-01 | | HLA-B | 7.4e-01 | 1.0e-00 | | PTPRN | 0.0e+00 | 1.0e-00 | | CPE | 0.0e+00 | 1.0e-00 | | HLA-DOA | 0.0e+00 | 1.0e-00 | | HLA-A | 0.0e+00 | 1.0e-00 | | HLA-C | 0.0e+00 | 1.0e-00 | | HLA-E | 0.0e+00 | 1.0e-00 | | HLA-G | 0.0e+00 | 1.0e-00 | |

  

---


Generated on: *Wed Apr 25 22:51:02 2007* - **R2HTML**


---
