# Supplementary material for: New insights about host response to smallpox using microarray data
Source: BMC Syst Biol. 2007 Aug 24;1:38. doi: 10.1186/1752-0509-1-38 (PMC2077868; doi:10.1186/1752-0509-1-38)
Supplement: Additional file 2 — Contribution of each molecule for the ED and LD active modules analysis. The contributions of individual molecules for each significantly altered module in the early and late death analysis. The contributions are represented by scores and the respective p-values. [file 1752-0509-1-38-S2.html]

 R output 


import namespace="mml" implementation="#mathplayer"?


**Alzheimers\_disease**

| |  | Score | P.valor | | --- | --- | --- | | LPL | 5.8e+00 | 7.6e-05 | | SNCA | 2.4e+00 | 1.9e-01 | | LRP1 | 1.4e+00 | 4.8e-01 | | APPBP1 | 9.8e-01 | 6.1e-01 | | GSK3B | 0.0e+00 | 8.7e-01 | | CASP3 | 0.0e+00 | 8.7e-01 | | APBB1 | 0.0e+00 | 8.7e-01 | | GAPD | 0.0e+00 | 8.7e-01 | |

  

**Cell\_adhesion\_molecules\_CAMs**

| |  | Score | P.valor | | --- | --- | --- | | ITGA2B | 8.2e+00 | 1.1e-07 | | ITGB5 | 6.1e+00 | 1.3e-04 | | SELP | 6.1e+00 | 1.3e-04 | | CD68 | 6.1e+00 | 1.3e-04 | | CD99 | 6.1e+00 | 1.3e-04 | | MCAM | 3.8e+00 | 2.2e-02 | | SDC3 | 3.8e+00 | 2.2e-02 | | HLA-DPA1 | 2.3e+00 | 1.9e-01 | | HLA-G | 2.1e+00 | 2.2e-01 | | HLA-DMA | 2.1e+00 | 2.2e-01 | | HLA-DPB1 | 2.1e+00 | 2.2e-01 | | HLA-DQB1 | 2.1e+00 | 2.2e-01 | | HLA-DQB2 | 2.1e+00 | 2.2e-01 | | HLA-DRA | 2.1e+00 | 2.2e-01 | | PLXNB2 | 2.0e+00 | 2.5e-01 | | HLA-DMB | 1.8e+00 | 2.8e-01 | | HLA-DQA2 | 1.8e+00 | 2.8e-01 | | CD2 | 0.0e+00 | 7.8e-01 | | CD48 | 0.0e+00 | 7.8e-01 | | ICAM1 | 0.0e+00 | 7.8e-01 | | ICAM2 | 0.0e+00 | 7.8e-01 | | ICAM3 | 0.0e+00 | 7.8e-01 | | HLA-A | 0.0e+00 | 7.8e-01 | | HLA-B | 0.0e+00 | 7.8e-01 | | HLA-C | 0.0e+00 | 7.8e-01 | | HLA-E | 0.0e+00 | 7.8e-01 | | HLA-F | 0.0e+00 | 7.8e-01 | | HLA-DOA | 0.0e+00 | 7.8e-01 | | HLA-DQA1 | 0.0e+00 | 7.8e-01 | | HLA-DRB3 | 0.0e+00 | 7.8e-01 | | ALCAM | 0.0e+00 | 7.8e-01 | | CD8A | 0.0e+00 | 7.8e-01 | | CD8B1 | 0.0e+00 | 7.8e-01 | | THY1 | 0.0e+00 | 7.8e-01 | | PECAM1 | 0.0e+00 | 7.8e-01 | | MADCAM1 | 0.0e+00 | 7.8e-01 | | ESAM | 0.0e+00 | 7.8e-01 | | ITGA2 | 0.0e+00 | 7.8e-01 | | ITGA3 | 0.0e+00 | 7.8e-01 | | ITGA5 | 0.0e+00 | 7.8e-01 | | ITGA6 | 0.0e+00 | 7.8e-01 | | ITGAV | 0.0e+00 | 7.8e-01 | | ITGAL | 0.0e+00 | 7.8e-01 | | ITGAE | 0.0e+00 | 7.8e-01 | | ITGB1 | 0.0e+00 | 7.8e-01 | | ITGB2 | 0.0e+00 | 7.8e-01 | | ITGB7 | 0.0e+00 | 7.8e-01 | | SELL | 0.0e+00 | 7.8e-01 | | CD44 | 0.0e+00 | 7.8e-01 | | CSPG2 | 0.0e+00 | 7.8e-01 | | CDH5 | 0.0e+00 | 7.8e-01 | | GLG1 | 0.0e+00 | 7.8e-01 | | SELPLG | 0.0e+00 | 7.8e-01 | | CD164 | 0.0e+00 | 7.8e-01 | | PLXNC1 | 0.0e+00 | 7.8e-01 | | NLGN1 | 0.0e+00 | 7.8e-01 | | PTPRC | 0.0e+00 | 7.8e-01 | |

  

**Cytokines**

| |  | Score | P.valor | | --- | --- | --- | | PPBP | 1.3e+01 | 1.4e-09 | | PF4 | 1.2e+01 | 1.4e-08 | | TNFSF10 | 5.5e+00 | 2.1e-02 | | GDF1 | 5.5e+00 | 2.1e-02 | | CCL4 | 4.6e+00 | 6.0e-02 | | IL24 | 2.1e+00 | 4.4e-01 | | TGFB2 | 2.1e+00 | 4.4e-01 | | GAS6 | 1.8e+00 | 5.0e-01 | | IL11 | 0.0e+00 | 8.5e-01 | | PRL | 0.0e+00 | 8.5e-01 | | IL10 | 0.0e+00 | 8.5e-01 | | VEGFB | 0.0e+00 | 8.5e-01 | | FLT3LG | 0.0e+00 | 8.5e-01 | | EFNA1 | 0.0e+00 | 8.5e-01 | | EFNA5 | 0.0e+00 | 8.5e-01 | | TNFSF7 | 0.0e+00 | 8.5e-01 | | TNFSF8 | 0.0e+00 | 8.5e-01 | | BMP8A | 0.0e+00 | 8.5e-01 | | INHBB | 0.0e+00 | 8.5e-01 | | CCL8 | 0.0e+00 | 8.5e-01 | | CCL3 | 0.0e+00 | 8.5e-01 | | CCL21 | 0.0e+00 | 8.5e-01 | | CCL5 | 0.0e+00 | 8.5e-01 | | CXCL13 | 0.0e+00 | 8.5e-01 | | IL8 | 0.0e+00 | 8.5e-01 | |

  

---


Generated on: *Wed Apr 25 22:51:21 2007* - **R2HTML**


---
