# Supplementary material for: New insights about host response to smallpox using microarray data
Source: BMC Syst Biol. 2007 Aug 24;1:38. doi: 10.1186/1752-0509-1-38 (PMC2077868; doi:10.1186/1752-0509-1-38)
Supplement: Additional file 4 — Relevance networks for module ECM receptor + CAMs of day0 vs. day4–6 analysis. Edges from the graphs in first page (day 0) and second page (days 4–6) represent significant (p < 10-3) negative (green) or positive (red) correlation values. In the last graph (third page), instead of correlation, the edges represent the p-values of the significantly changes of the correlations. [file 1752-0509-1-38-S4.pdf]

ECM receptors + CAMs Day 0

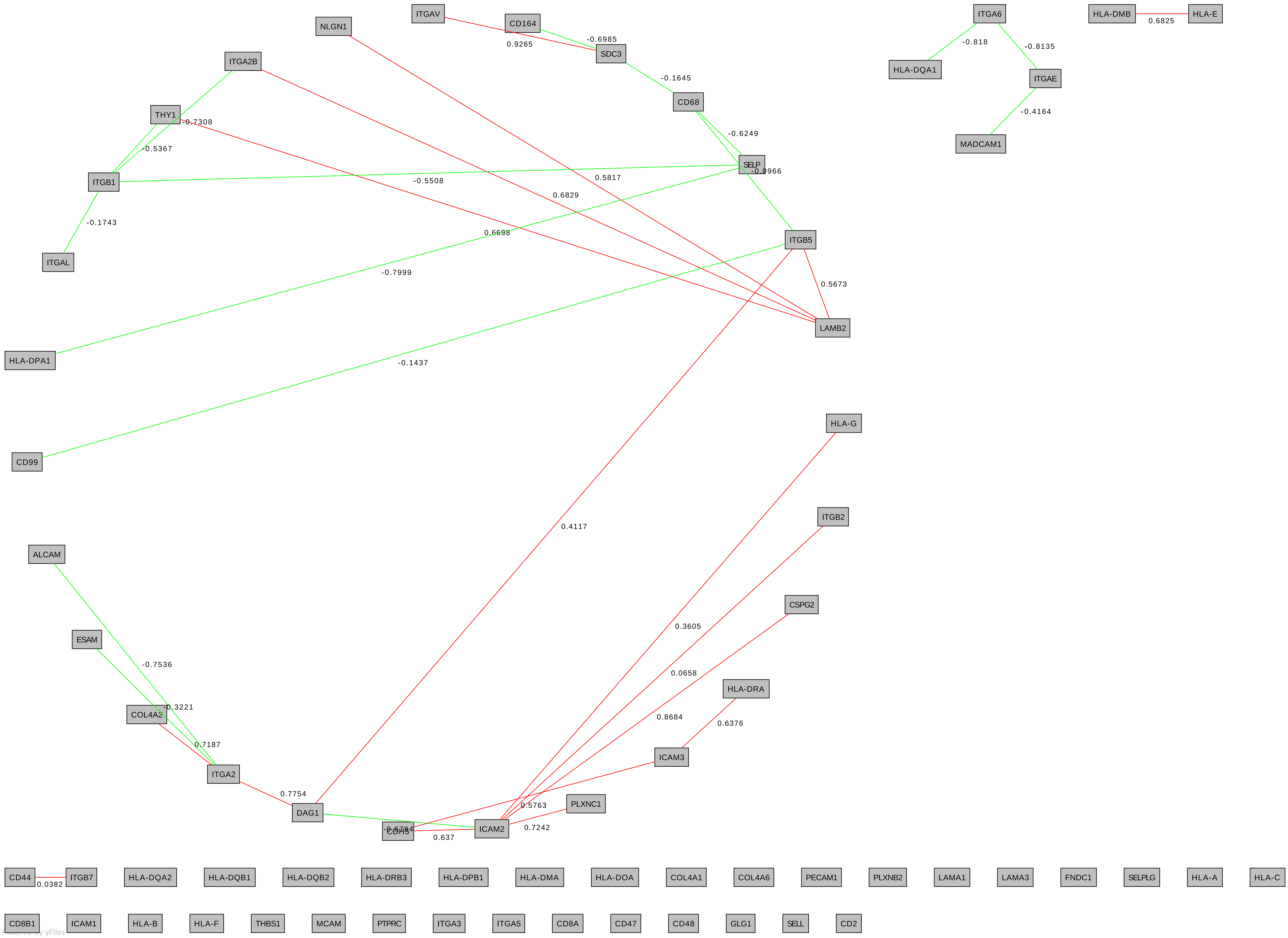

ECM receptors + CAMs Days 4 up to 6

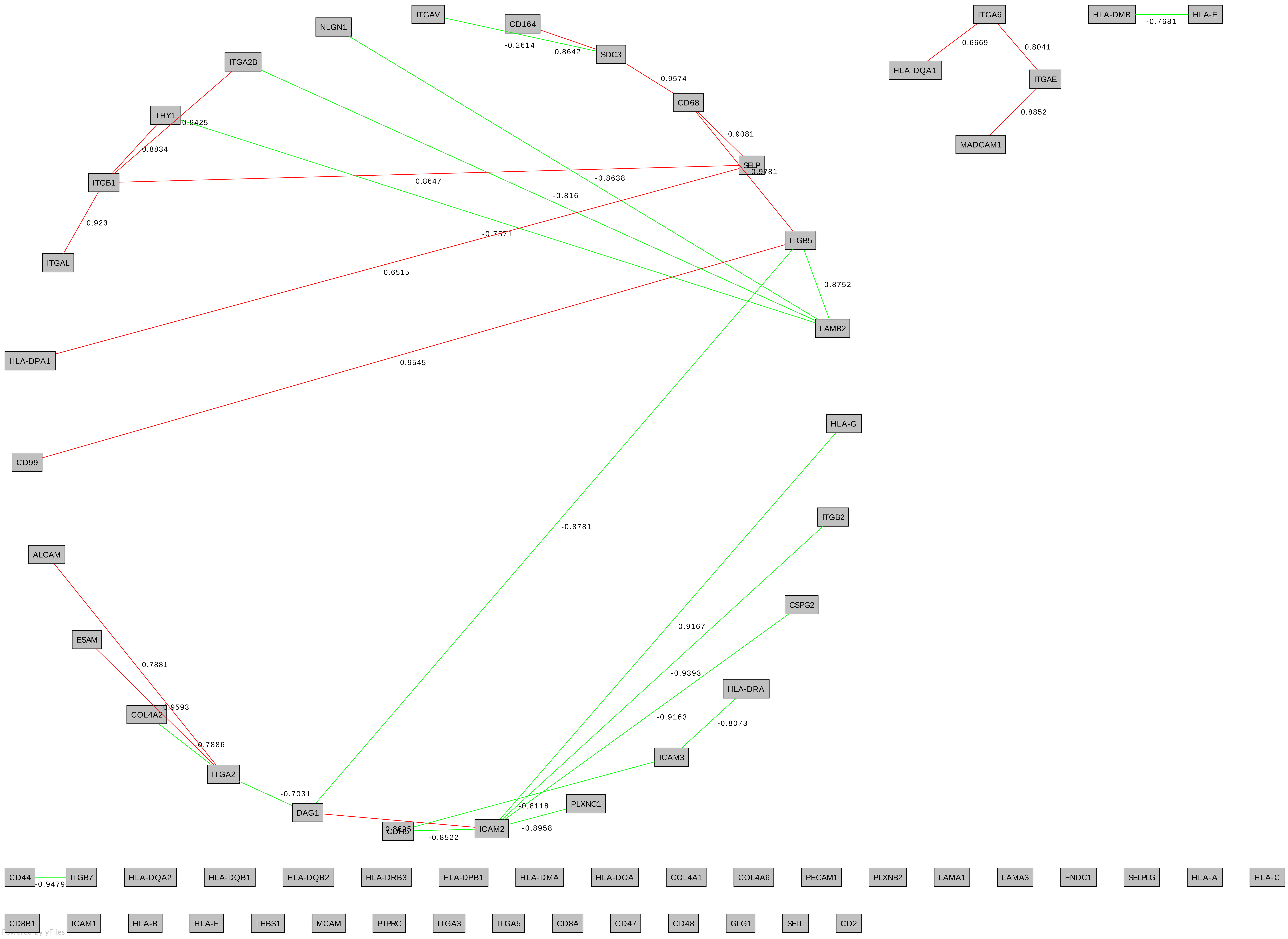

ECM receptors + CAMs P-values

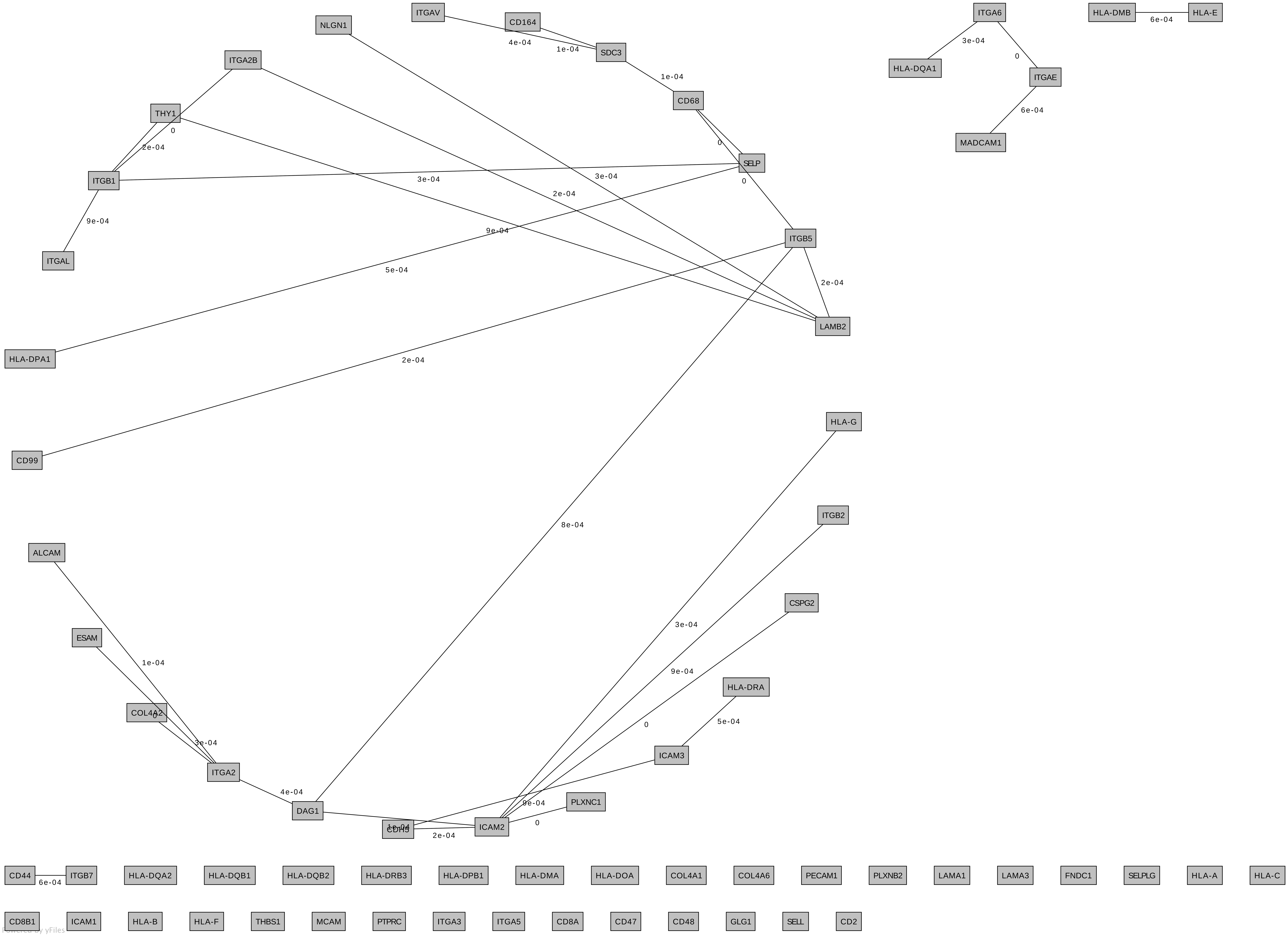

Powered by yFiles

Additional file 4: Relevance networks for module ECM receptors + CAMs.
Edges from the graphs in first page (day 0) and second page (days 4-6) represent significant ($p < 10^{-3}$) negative (green) or positive (red) correlation values.
In the last graph (third page), instead of correlation, the edges represent the p-values of the significantly changes of the correlations.
