# Supplementary material for: New insights about host response to smallpox using microarray data
Source: BMC Syst Biol. 2007 Aug 24;1:38. doi: 10.1186/1752-0509-1-38 (PMC2077868; doi:10.1186/1752-0509-1-38)
Supplement: Additional file 5 — Relevance networks for module Cytokines of day0 vs. day1–3. Edges from the graphs in first page (day 0) and second page (days 1–3) represent significant (p < 10-3) negative (green) or positive (red) correlation values. In the last graph (third page), instead of correlation, the edges represent the p-values of the significantly changes of the correlations. For this module the comparison of day 0 vs. days 4–6 has no statistically significant results. [file 1752-0509-1-38-S5.pdf]

Cytokines Day 0

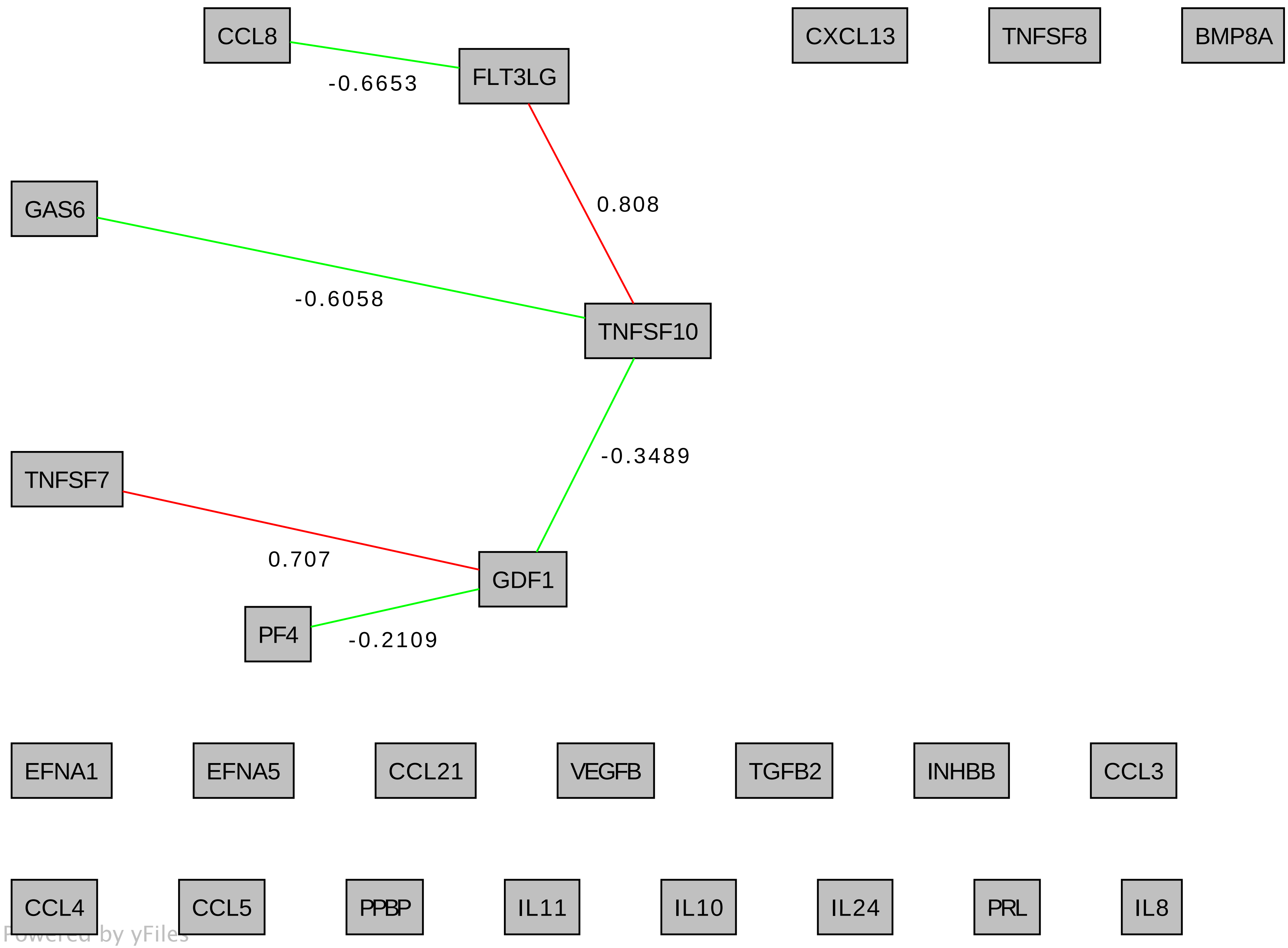

Cytokines Days 1 up to 3

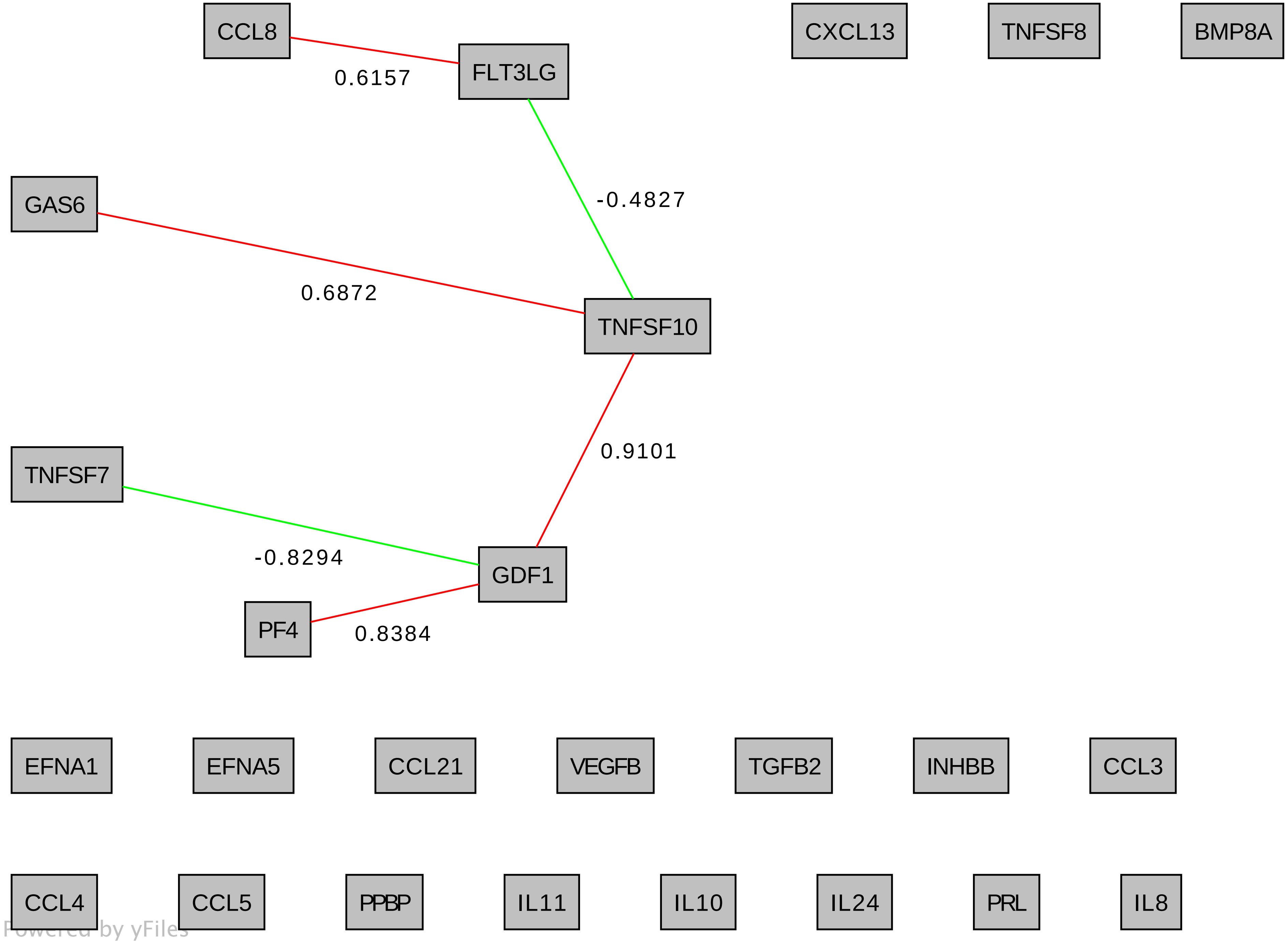

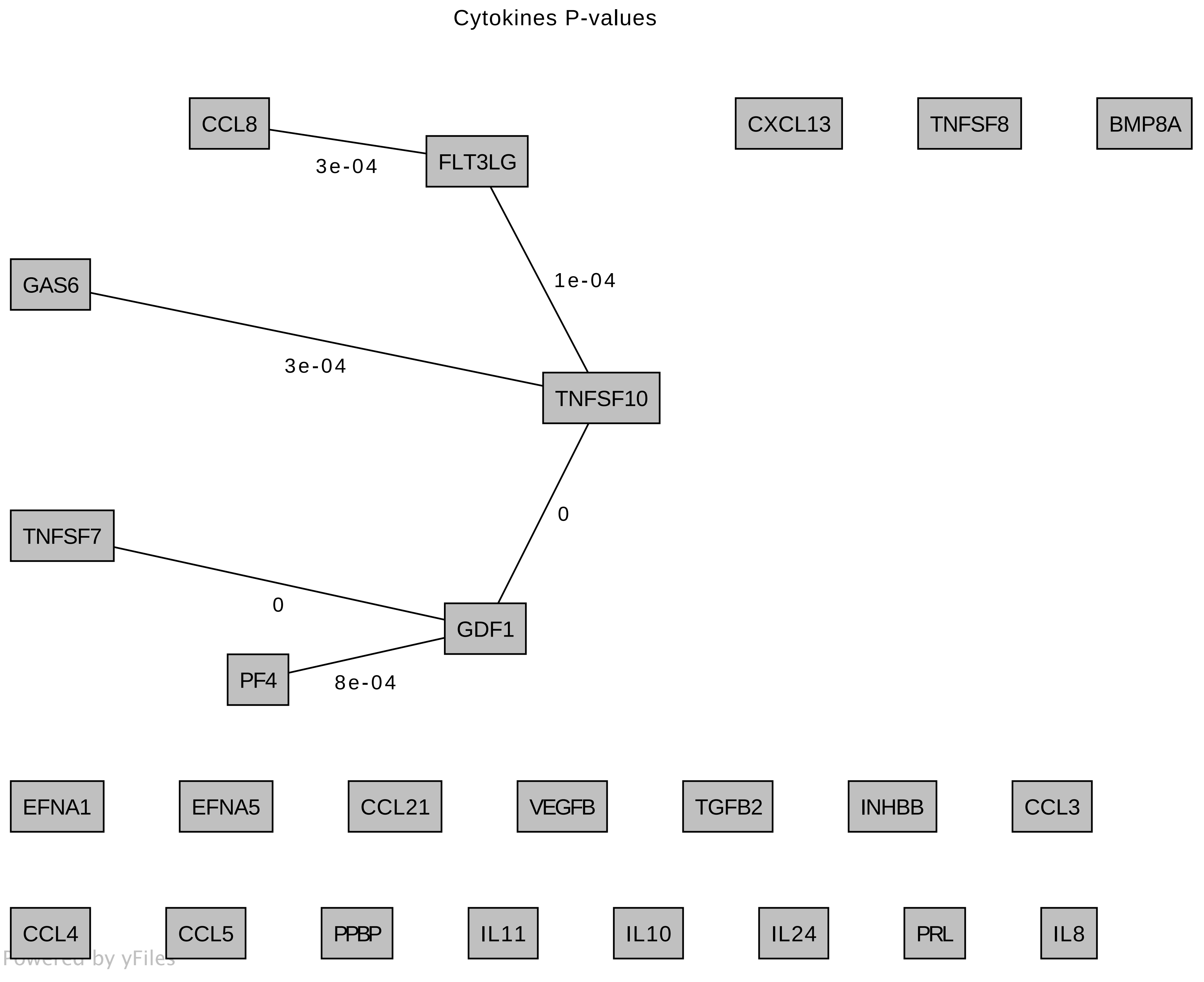
Cytokines P-values
CCL8
FLT3LG
3e-04
CXCL13
TNFSF8
BMP8A
GAS6
1e-04
3e-04
TNFSF10
TNFSF7
0
0
GDF1
PF4
8e-04
EFNA1
EFNA5
CCL21
VEGFB
TGFB2
INHBB
CCL3
CCL4
CCL5
PPBP
IL11
IL10
IL24
PRL
IL8
Powered by yFiles

Additional file 5: Relevance networks for module Cytokines.
Edges from the graphs in first page (day 0) and second page (days 1-3) represent significant ($p < 10^{-3}$) negative (green) or positive (red) correlation values.
In the last graph (third page), instead of correlation, the edges represent the p-values of the significantly changes of the correlations.
For this module the comparison of day 0 vs days 4-6 has no statistically significant results.
